# Associations between serum electrolyte and short-term outcomes in patients with acute decompensated heart failure

**DOI:** 10.1080/07853890.2022.2156595

**Published:** 2022-12-15

**Authors:** Kai Zhao, Qun Zheng, Jiang Zhou, Qi Zhang, Xiaoli Gao, Yinghua Liu, Senlin Li, Weichao Shan, Li Liu, Nan Guo, Hongsen Tian, Qingmin Wei, Xitian Hu, Yingkai Cui, Xue Geng, Qian Wang, Wei Cui

**Affiliations:** a Department of Cardiology, The Second Hospital of Hebei Medical University, Shijiazhuang, China; b Department of Cardiology, Hengshui People’s Hospital, Hengshui, China; c Department of Cardiology, Chengde Central Hospital, Chengde, China; d Department of Cardiology, First Central Hospital of Baoding, Baoding, China; e Department of Cardiology, Huabei Petroleum Administration Bureau General Hospital, Cangzhou, China; f Department of Cardiology, First Hospital of Zhangjiakou, Zhangjiakou, China; g Department of Cardiology, Affiliated Hospital of Chengde Medical University, Chengde, China; h Department of Cardiology, First Hospital of Qinhuangdao, Qinhuangdao, China; i Department of Cardiology, Cangzhou Central Hospital, Cangzhou, China; j Department of Cardiology, Handan Central Hospital, Handan, China; k Department of Cardiology, Xingtai People’s Hospital, Xingtai, China; l Department of Cardiology, Shijiazhuang People’s Hospital, Shijiazhuang, China; m Department of Cardiology, The 252nd Hospital of People’s Liberation Army, Baoding, China

**Keywords:** Serum potassium, serum sodium, serum chloride, serum total calcium, short-term prognosis, acute decompensated heart failure

## Abstract

**Introduction:**

There is a dearth of comprehensive studies on the association between serum electrolyte and adverse short-term prognosis of Chinese patients with acute decompensated heart failure (ADHF).

**Patients and methods:**

A total of 5166 patients with ADHF were divided into four serum electrolyte-related study populations (potassium (*n* = 5145), sodium (*n* = 5135), chloride (*n* = 4966), serum total calcium (STC) (*n* = 4143)) under corresponding exclusions. Different logistic regression models were utilized to gauge the association between these electrolytes or the number of electrolyte abnormalities and the risk of a composite of all-cause mortality or 30-day heart failure (HF) readmission.

**Results:**

In multivariable adjusted analysis, patients with potassium below 3.5 mmol/L (odds ratios (ORs) 1.45; 95% confidence interval (CI):1.07–1.95), 4.01–4.50 mmol/L (OR: 1.29, CI: 1.02–1.62), 4.51–5.00 mmol/L (OR: 1.43, CI: 1.08–1.90) and above 5.00 mmol/L (OR: 1.74, CI: 1.21–2.51) had an increased risk of outcome when compared with potassium at 3.50–4.00 mmol/L. Sodium levels were inversely related to the risk of a composite outcome (<130 mmol/L: OR: 2.73 (95% CI, 1.81–4.12); 130–134 mmol/L: OR, 1.97 (CI, 1.45–2.68); 135–140 mmol/L: OR, 1.45 (CI, 1.17–1.81); *p* for trend < 0.001) in comparison with sodium at 141–145 mmol/L. Chloride < 95 mmol/L corresponded to a higher risk of a composite outcome with an OR of 1.65 (95% CI, 1.16–2.37) in contrast to chloride levels at 101–105 mmol/L. In addition, the adjusted ORs (95% CI) for a composite outcome comparing the STC < 2.00 and 2.00–2.24 vs. 2.25–2.58 mmol/L were 0.98 (0.69–1.43) and 1.13 (0.89–1.44), respectively. Besides that, the number of electrolyte abnormalities was positively related to the risk of a composite outcome (*N* = 1, OR 1.40, 95% CI: 1.13–1.73; *N* = 2, OR 2.51, 95% CI: 1.85–3.42; *N* = 3, OR 2.47, 95% CI: 1.45–4.19; *p* for trend < 0.001) in comparison with *N* = 0.

**Conclusions:**

A deviation of potassium levels from 3.50 to 4.00 mmol/L, lower sodium levels and hypochloremia were associated with poorer short-term prognosis of ADHF. Furthermore, the number of electrolyte abnormalities positively correlated with adverse short-term prognosis of patients with ADHF.

**Key Messages**
- ADHF patients with baseline serum potassium at first half part of normal range (3.50–4.00 mmol/L) may herald the lowest risk of recent cardiovascular events.
- Serum sodium and chloride levels exhibit discrepancies in terms of risk of short-term adverse events of ADHF patients.
- The number of electrolyte abnormalities is a significant predictor of poor short-term prognosis in patients with ADHF.

**Clinical trial registration URL:**

http://www.chictr.org.cn/showproj.aspx?proj=23139. Unique identifier: ChiCTR-POC-17014020.

## Introduction

Acute decompensated heart failure (ADHF) is defined as the new onset or recurrence of deteriorating signs or symptoms of heart failure (HF) requiring unscheduled medical care or hospitalization, which renders it as one of the most common causes for hospitalization in older patients globally. In China, the epidemiologic data indicate that about 13.7 million people are living with HF, with its incidence still on an upward trend [1]. There is no globally accepted classification of ADHF; however, it can be defined mostly as new-onset and decompensated HF [2]. According to recent clinical studies, ADHF is known to have a relatively low in-patient mortality, yet it can lead to a high rate of recurrent post-discharge events, especially within three months [3–8]. In addition, latest evidence has manifested only a slight impact on rehospitalizations despite substantial efforts to decrease the readmission rate for ADHF [9]. Therefore, factors influencing the short-term prognosis of ADHF should become a major focus for clinicians or researchers.

In general, patients with ADHF are prone to face electrolyte disorders, which result due to variations in levels of potassium (hyperkalaemia, hypokalaemia), sodium (hypernatremia, hyponatremia) and calcium (hypercalcemia, hypocalcaemia). This may be caused due to a disturbance of renin–angiotensin–aldosterone and sympathetic nervous system, as well as a consequence of diuretic therapy and comorbidity burden (e.g. poor renal function). Furthermore, ion disturbances play a significant role in the development of HF, thereby imposing a considerable influence on the medical therapies and prognosis of patients with ADHF. Recent studies have shown a U-shaped relationship between potassium levels and mortality in both patients with AHF and chronic heart failure (CHF) [10,11]. According to previous studies, hyponatremia (major cation abnormality) is closely related to undesirable short- and long-term prognosis of patients with HF; increase in its severity leads to a greater incidence of adverse events [12–14]. Hypochloremia (anion disturbance) has been associated with adverse prognosis in ADHF, as chloride mainly exists as sodium chloride in the extracellular fluid [15–17]. Additionally, previous studies have also reiterated that hypocalcaemia (mostly assessed by ionized calcium) is linked to poor prognosis of patients with HF [18,19]. Administration of serum total calcium (STC) is preferred over ionized calcium in the clinical practice performed in medical facilities, ranging from community clinics, school infirmaries to tertiary hospitals, across China. But above all, only a few researchers have conducted studies on the relationship between the number of electrolyte disorders and poor short-term of prognosis of patients with ADHF.

Therefore, we performed a retrospective analysis to gauge the relevance of four common electrolytes (potassium, sodium, chloride and STC) together with the number of electrolyte abnormalities in the poor short-term prognosis of patients with ADHF.

## Patients and methods

### Study population

The Heb-ADHF (Hebei-acute decompensated heart failure) study (ChiCTR-POC-17014020) is a prospective, multicentre and observational study, conducted on patients discharged from hospitals with a main diagnosis of HF in accordance with the Chinese HF guideline [2]. The study was approved by the Human Research Ethics Committee of the Second Hospital of Hebei Medical University (2015110) and is in conformity with the ethical standards of the institutional and/or national research committee and 1964 Helsinki Declaration as well as its later amendments or comparable ethical standards. Informed consents were obtained from all patients included in the study.

#### Ethics, consent and permissions

Verbal informed consents were obtained from all patients included in the study.

#### Consent to publish

We have obtained consent to publish from the participant to report individual patient data.

ADHF was defined as a de novo AHF or decompensation of CHF. Inclusion criteria for the study were: (1) age ≥18 y; (2) unplanned admission; (3) typical symptoms or signs of ADHF; and (4) brain natriuretic peptide (BNP) levels > 100 pg/mL or N-terminal pro-brain natriuretic peptide (NT-proBNP) levels > 300 pg/mL. Exclusion criteria for the study were: (1) hospitalization < 24 h; (2) heart transplantation; (3) ongoing renal replacement therapy; (4) massive stroke; (5) concomitant terminal disease; or (6) patients lost to follow-up. Furthermore, patients with history of parathyroid disease or vitamin D-related disorders were excluded from the study.

We gathered data on the baseline characteristics of the patients as follows: age, sex, body mass index (BMI, calculated by dividing weight (in kg) by height (in m) squared), details of first admission, smoking and drinking status, New York Heart Association (NYHA) or Killip functional class (if both grading methods were applicable to a patient with acute myocardial infarction (AMI); the more severe one was chosen), comorbidities compromising coronary artery disease (CAD), AMI, hypertension, valvular heart disease (VHD), dilated cardiomyopathy (DCM), diabetes mellitus (DM), stroke/transient ischemic attack (TIA), atrial fibrillation/atrial flutter (A fib/A flutter), chronic kidney disease (CKD) as well as records of physical examinations, laboratory tests and baseline medications. Each variable was based on the strength of the European Society of Cardiology (ESC) recommendations on HF initial evaluation [20].

**Figure 1.**
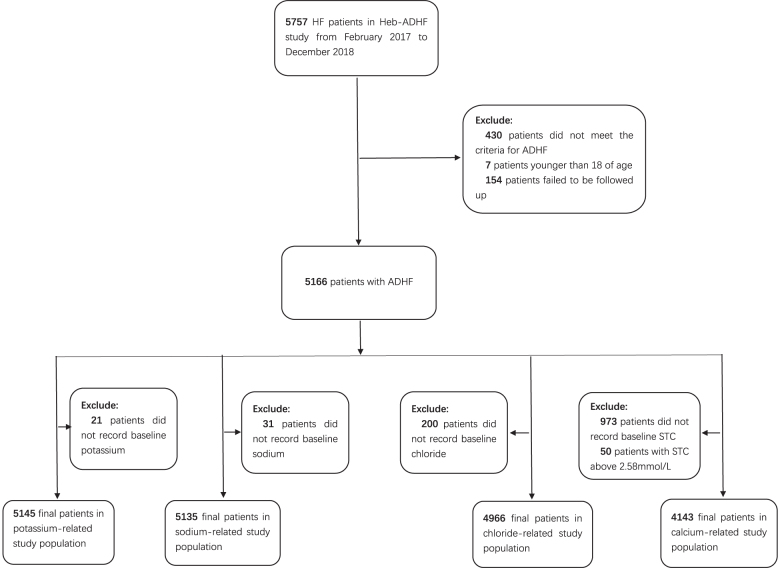
Total Heb-ADHF population; exclusions and the final study population. Heb-ADHF: Hebei-acute decompensated heart failure; STC: serum total calcium; ADHF: acute decompensated heart failure.

In order to explore the correlation between serum electrolyte levels and adverse events in patients with ADHF, participants were chosen as the four serum electrolyte-related study populations via corresponding exclusions from the Heb-ADHF Registry. Furthermore, the Cl^–^-related study population was used to gauge the association between the number of electrolyte disorders and the adverse prognosis of ADHF. A flowchart describing further details of patient selection is shown in Figure 1.

### Pre-defined serum electrolyte intervals and the number of electrolyte abnormalities

Blood samples were collected at the time of admission and analysed immediately. The normal ranges for serum electrolyte concentrations (potassium: 3.5–5.0 mmol/L, sodium: 135–145 mmol/L, chloride: 95–105 mmol/L and STC: 2.25–2.58 mmol/L), were considered in accordance with the latest guidelines [21]. In the potassium-related study population, baseline characteristics were compared among five groups of patients as per the varying potassium levels: <3.50, 3.50–4.00, 4.01–4.50, 4.51–5.00 and >5.00 mmol/L. Interval 2 (3.50–4.00 mmol/L) was reckoned as a reference for statistical analysis. Hypokalaemia is defined as potassium < 3.5 mmol/L and hyperkalaemia as >5.0 mmol/L. The other three study populations were treated similarly as well. Specific details are described in Table 1. Electrolyte abnormalities were defined as K^+^<3.5 or >5.0, Na^+^<135 and Cl^–^<95 mmol/L; Therefore, the maximal number of electrolyte abnormalities was 3, in terms of each individual participant.

**Table 1.** Pre-defined serum electrolytes concentration intervals.

Serum electrolyte concentration (mmol/L)					
Potassium	Interval 1 (hypokalemia) <3.50	Interval 2 (Ref.) 3.50–4.00	Interval 3 4.01–4.50	Interval 4 4.51–5.00	Interval 5 (hyperkalemia) >5.00
Sodium	Interval 1 <130	Interval 2 (hyponatremia) 130–134	Interval 3 135–140	Interval 4 (Ref.) 141–145	Interval 5 (hypernatremia) >145
Chloride	Interval 1 (hypochloremia) <95	Interval 2 95–100	Interval 3 (Ref.) 101–105	Interval 4 (hyperchloremia) >105	
STC	Interval 1 <2.00	Interval 2 (hypocalcemia) 2.00–2.24	Interval 3 (Ref.) 2.25–2.58		

STC: serum total calcium.

### Follow-up and outcomes

Each recruited patient was followed up at 30 days after discharge via telephonic interview by trained staff. Information on events of readmission or death was gathered at each follow-up. The loss ratio of follow-up was around 2.7%. The primary outcome was a composite of short-term (in-patient or within 30 days after discharge) all-cause mortality or 30-day HF readmission.

### Statistical analysis

Baseline characteristics were presented in each interval of electrolytes. The Kolmogorov–Smirnov (*n* ≥ 5000) or Shapiro–Wilk (*n* < 5000) test was performed to assess the distribution of quantitative variables. The normally distributed and homogenous data were reported as mean ± standard deviation (SD), followed by evaluation of differences among the distinct groups in each study population by one-way analysis of variance (ANOVA). Contrarily, variables were described as medium and interquartile range (IQR). In addition, the Kruskal–Wallis test was applied for the assessment of discrepancies in measurement data among the different groups. Categorical variables were presented as numbers and percentages (%) and analysed by Pearson’s Chi-square test or Fisher’s exact test. Pairwise comparisons were performed through Bonferroni’s corrections.

Baseline variables that were deemed clinically related or that displayed a univariable relevance to the risk of a composite outcome with a *p* value < 0.2 were entered into the multivariable logistic regression models (further details are shown in Supplementary online resource Table 1). Variables for inclusion were deliberately chosen, considering the number of events available, to ensure parsimony of the final models.

Multivariable logistic regression models were performed to eliminate the impact of confounding factors; while sensitivity analyses were implemented to ensure data robustness. Odds ratios (ORs) and 95% confidence intervals (CIs) were calculated for determining the association between serum electrolyte levels and the risk of a primary composite outcome via logistic regression models. Each quantitative variable was verified to have a linear relationship with logit transformation on outcome by means of the Box–Tidwell method. Based on the results of the variance inflation factors, multi-collinearity did not exist among all the confounders (VIF, all < 10, correlation coefficients between each two variables were less than 0.7). Multivariable models were first adjusted for age and sex (model 1), and were then further adjusted for first admission, smoking, drinking, NYHA or Killip functional class, comorbidities including CAD, AMI, hypertension, DCM, A fib/A flutter, CKD and corresponding variables among the four study populations (model 2). The models were further adjusted for confounders with respect to physical examinations (heart rate, systolic blood pressure, etc.), and laboratory tests, as well as baseline medications (model 3, fully adjusted model). Additionally, we conducted tests for analysing the linear trends by entering the median value of each interval of electrolyte concentration as a continuous variable in the models.

To maximize the statistical power and minimize bias, patients with missing data were not excluded from analyses. We used multiple imputation (MI), based on five replications and the Markov Chain Monte Carlo method in the SPSS MI procedure, to account for the missing data on serum albumin, left ventricular ejection fraction (LVEF) and estimated glomerular filtration rate (eGFR) [22]. BNP and NT-pro BNP were not considered for MI, given the large amount of fragmentary data. Finally, we created a variable log (BNP or NT-pro BNP) that indicated ‘natriuretic peptide’, which was combined with the log-transformed BNP and NT-pro BNP data, giving rise to all the missing data that were completely imputed according to the different electrolyte-related study populations. The Supplementary online resource Table 2 gives additional details of the statistical analyses.

We did subgroup analyses by perfusion status which provides important information on the HF management according to previous studies [23,24]. The potential hypoperfusion group was defined as a composite of narrow proportional pulse pressure ((SBP – DBP)/SBP)<25%, SBP < 80 mmHg, heart rate/SBP ≥ 1 together with sinus rhythm, or Killip class IV considering the limited data we gathered (e.g. absence of clinical profiles with regard to cool extremities, impaired mentation or oliguria), while the rest of participants were classified as adequate perfusion group. In addition, the interactions between perfusion status and serum electrolyte were tested. We also performed sensitivity analyses to ensure data robustness via the following steps: not imputed and multiple imputed serum albumin, LVEF, eGFR and log (BNP or NT-pro BNP) data were fed into fully adjusted models (model 3) for further adjustment as each of them showed a univariable relevance to the risk of a composite outcome with a *p* value <0.2. Additionally, patients with NYHA II were excluded. The data of the remaining participants were used in the aforementioned models for reassessment. Multivariable logistic regression models were identically implemented in sensitivity analyses.

All analyses were performed using the SPSS version 25 (SPSS Inc., Chicago, IL). A two-sided *p* value < 0.05 was considered to be statistically significant.

## Results

A total of 5145 participants (58.3% men; median age 69 y) comprised the potassium-related study population, with 631, 1784, 1726, 720 and 284 patients classified as internal 1 (K: <3.50 mmol/L), internal 2 (K: 3.50–4.00 mmol/L), interval 3 (K: 4.01–4.50 mmol/L), interval 4 (K: 4.51–5.00 mmol/L) and interval 5 (K: >5.00 mmol/L), respectively. Meanwhile, the sodium-related study population (58.3% men; median age 69 y) included 5135 participants categorized into five groups according to their serum sodium levels. Furthermore, 4966 patients from the chloride-related study population (58.0% men; median age 69 y) were divided into four different groups based on their serum chloride concentrations. Finally, differing from the above, the STC-related study population (58.7% men; median age 68 y) was composed of 4143 patients stratified into three groups on the basis of their STC levels.

### Characteristics of the study participants

On the whole, patients without electrolyte abnormalities tended to be younger and had a higher proportion of male and first admission, lower prevalence of AMI, DM and CKD, increased level of SBP, DBP and serum albumin, decreased concentration of BNP or NT-pro BNP, as well as more frequency usage of angiotensin converting enzyme-1/angiotensin receptor blockers (ACEI/ARBs) or β-blockers than participants with electrolyte disturbances. Further details and other baseline characteristics of different study populations are summarized in Table 2 and Supplementary online resource Tables 3–6.

**Table 2.** Baseline characteristics concerning four groups of participants classified by the number of electrolyte abnormalities.

Characteristics	Level	0 (*n* = 3465)	1 (*n* = 1135)	2 (*n* = 283)	3 (*n* = 83)	*p* Value
Age (years)	Median (IQR)	68 (60–76)	70 (61–78)	73 (63–80)	68 (58–76)	<0.001
Older people (≥65 years)	*n* (%)	2147 (62.0)	756 (66.6)	202 (71.4)	53 (63.9)	0.001
Male	*n* (%)	2401 (58.9)	651 (57.4)	151 (53.4)	37 (44.6)	0.019
BMI (kg/m^2^)	Median (IQR)	24.5 (22.2–27.0)	24.2 (21.6–26.4)	23.8 (20.8–26.6)	23.4 (20.6–25.5)	<0.001
Missing	*n* (%)	792 (22.9)	312 (27.5)	99 (35.0)	29 (34.9)	
First admission	*n* (%)	1841 (53.1)	542 (47.8)	101 (35.7)	23 (27.7)	<0.001
Smoking	*n* (%)	875 (25.3)	255 (22.5)	51 (18.0)	13 (15.7)	0.004
Drinking	*n* (%)	738 (21.3)	230 (20.3)	46 (16.3)	9 (10.8)	0.027
NYHA class	*n* (%)					<0.001
II		398 (11.5)	78 (6.9)	8 (2.8)	3 (3.6)	
III		1106 (31.9)	288 (25.4)	76 (26.9)	12 (14.5)	
IV		1417 (40.9)	553 (48.7)	153 (54.1)	61 (73.5)	
Killip class						
II		364 (10.5)	146 (12.9)	29 (10.2)	4 (4.8)	
III		125 (3.6)	38 (3.3)	12 (4.2)	3 (3.6)	
IV		55 (1.6)	32 (2.8)	5 (1.8)	0 (0)	
Comorbidity	*n* (%)					
CAD		1854 (53.5)	668 (58.9)	160 (56.5)	30 (36.1)	<0.001
AMI		652 (18.8)	261 (23.0)	56 (19.8)	10 (12.0)	0.006
Hypertension		2023 (58.4)	681 (60.0)	142 (50.2)	39 (47.0)	0.004
VHD		500 (14.4)	156 (13.7)	51 (18.0)	21 (25.3)	0.012
DCM		484 (14.0)	143 (12.6)	43 (15.2)	17 (20.5)	0.170
DM		866 (25.0)	338 (29.8)	76 (26.9)	25 (30.1)	0.013
Stroke/TIA		590 (17.0)	224 (19.7)	55 (19.4)	10 (12.0)	0.082
A fib/A flutter		996 (28.7)	317 (27.9)	89 (31.4)	33 (39.8)	0.101
CKD		190 (5.5)	93 (8.2)	20 (7.1)	15 (18.1)	<0.001
Physical examinations	Median (IQR)					
Heart rate (bpm)		84 (70–99)	84 (72–100)	86 (72–102)	78 (67–102)	0.079
SBP (mmHg)		130 (117–147)	130 (114–150)	122 (109–140)	113 (102–134)	<0.001
DBP (mmHg)		80 (70–90)	79 (70–90)	73 (65–86)	70 (62–84)	<0.001
LVEF (%)		45 (37–57)	44 (35–56)	44 (34–56)	44 (33–57)	0.002
Missing	*n* (%)	83 (2.4)	24 (2.1)	12 (4.2)	2 (2.4)	
Laboratory tests	Median (IQR)					
BNP (pg/mL)		649 (287–1360)	917 (393–1742)	971 (423–2018)	1180 (698–2180)	<0.001
Missing	*n* (%)	1244 (35.9)	439 (38.7)	117 (41.3)	36 (43.4)	
NT-proBNP (pg/mL)		3902 (1895–7521)	5057 (2350–9867)	6597 (2442–12,960)	7315 (3464–15,039)	<0.001
Missing	*n* (%)	2340 (67.5)	736 (64.8)	171 (60.4)	53 (63.9)	
eGFR (mL/min/1.73 m^2^)		81 (63–101)	75 (53–96)	65 (47–89)	64 (38–98)	<0.001
Missing	*n* (%)	40 (1.2)	20 (1.8)	5 (1.8)	1 (1.2)	
FBG (mmol/L)		5.6 (4.8–7.0)	5.9 (5.0–7.9)	6.2 (5.2–8.7)	5.9 (5.0–8.4)	<0.001
Missing	*n* (%)	261 (7.5)	104 (9.2)	32 (11.3)	10 (12.0)	
Serum albumin (g/L)		39 (36–42)	38 (34–41)	38 (35–41)	36 (33–40)	<0.001
Missing	*n* (%)	96 (2.8)	42 (37)	17 (6.0)	4 (4.8)	
Potassium (mmol/L)		4.1 (3.8–4.4)	3.7 (3.4–4.5)	4.1 (3.6–4.7)	3.4 (3.3–5.4)	<0.001
Sodium (mmol/L)		140 (138–142)	138 (134–142)	132 (128–134)	130 (127–133)	<0.001
Chloride (mmol/L)		103 (101–106)	101 (98–104)	93 (90–95)	90 (86–93)	<0.001
Baseline medications	*n* (%)					
Loop diuretics		3002 (86.6)	1030 (90.7)	261 (92.2)	73 (88.0)	<0.001
Thiazide diuretics		327 (9.4)	91 (8.0)	26 (9.2)	7 (8.4)	0.545
ACEI/ARBs		1889 (54.5)	583 (51.4)	131 (46.3)	35 (42.2)	0.004
β-blockers		2416 (69.7)	737 (64.9)	177 (62.5)	52 (62.7)	0.002
MRA (spironolactone)		2833 (81.8)	952 (83.9)	232 (82.0)	76 (91.6)	0.057
Digitalis		789 (22.8)	274 (24.1)	77 (27.2)	26 (31.3)	0.099
Nitrates		1710 (49.4)	600 (52.9)	127 (44.9)	32 (38.6)	0.009
Levosimendan		158 (4.6)	48 (4.2)	14 (4.9)	7 (8.4)	0.337
Nesiritide		262 (7.6)	114 (10.0)	35 (12.4)	11 (13.3)	0.002
Statin		2358 (68.1)	775 (68.3)	165 (58.3)	40 (48.2)	<0.001

BMI: body mass index; NYHA: New York Heart Association; CAD: coronary artery disease; AMI: acute myocardial infarction; VHD: valvular heart disease; DCM: dilated cardiomyopathy; DM: diabetes mellitus; TIA: transient ischemic attack; A fib/A flutter: atrial fibrillation/atrial flutter; CKD: chronic kidney disease; SBP: systolic blood pressure; DBP: diastolic blood pressure; LVEF: left ventricular ejection fraction; BNP: brain natriuretic peptide; NT-pro BNP: N-terminal pro brain natriuretic peptide; eGFR: estimated glomerular filtration rate; FBG: fasting blood glucose; ACEI/ARBs: angiotensin-converting enzyme inhibitors/ angiotensin receptor blockers; MRA: mineralocorticoid receptor antagonist.

Percentages may not total 100 because of rounding.

### Relationship between serum electrolytes and poor short-term prognosis of ADHF

During a 30-day follow-up on each discharged patient, the number of outcome events spotted in the potassium, sodium, chloride and STC-linked populations was 562, 561, 549 and 444, respectively. The in-patient all-cause mortality of each study population reached an estimate of 4.2%, slightly higher than that of 3% reported in a previous study [25]. Meanwhile, the incidence of HF rehospitalizations at 30 days for each population was roughly 5.3%, which basically mirrors the result of a former trial [26]. Detailed information is described in Table 3.

**Table 3.** Short-term outcomes in participants according to different study populations.

K^+^-related study population	All (5145), *n* (%)	Interval 1 (*n* = 631)	Interval 2 (*n* = 1784)	Interval 3 (*n* = 1726)	Interval 4 (*n* = 720)	Interval 5 (*n* = 284)	*p* Value
All-cause mortality	287 (5.6)	39 (6.2)	74 (4.1)	96 (5.5)	49 (6.7)	29 (10.2)	<0.001
In-hospital	211 (4.1)	30 (4.8)	57 (3.2)	71 (4.1)	34 (4.7)	19 (6.7)	0.041
30-day post-discharge	76 (1.5)	9 (1.4)	17 (0.9)	25 (1.4)	15 (2.0)	10 (3.5)	0.014
All-cause 30-day readmission	397 (7.8)	51 (8.1)	120 (6.8)	136 (7.9)	61 (8.5)	29 (10.3)	0.223
HF	275 (5.3)	38 (6.0)	76 (4.3)	93 (5.4)	44 (6.1)	24 (8.5)	0.026
Non-HF	122 (2.4)	13 (2.1)	44 (2.5)	43 (2.5)	17 (2.4)	5 (1.8)	0.925

Na^+^-related study population	All (5135), *n* (%)	Interval 1 (*n* = 170)	Interval 2 (*n* = 481)	Interval 3 (*n* = 2378)	Interval 4 (*n* = 1831)	Interval 5 (*n* = 275)	*p* Value
All-cause mortality	286 (5.6)	21 (12.9)	52 (10.8)	137 (5.8)	66 (3.6)	9 (3.3)	<0.001
In-hospital	210 (4.1)	15 (8.8)	37 (7.7)	99 (4.2)	53 (2.9)	6 (2.2)	<0.001
30-day post-discharge	76 (1.5)	7 (4.1)	15 (3.1)	38 (1.6)	13 (0.7)	3 (1.1)	<0.001
All-cause 30-day readmission	396 (7.8)	22 (13.0)	47 (9.8)	203 (8.6)	112 (6.1)	12 (4.4)	<0.001
HF	275 (5.4)	19 (11.2)	34 (7.1)	144 (6.1)	70 (3.8)	8 (2.9)	<0.001
Non-HF	121 (2.4)	3 (1.8)	13 (2.7)	59 (2.5)	42 (2.3)	4 (1.5)	0.851

Cl^–^-related study population	All (4966), *n* (%)	Interval 1 (*n* = 381)	Interval 2 (*n* = 1297)	Interval 3 (*n* = 1964)	Interval 4 (*n* = 1324)	*p* Value
All-cause mortality	280 (5.6)	49 (12.9)	93 (7.2)	77 (3.9)	61 (4.6)	<0.001
In-hospital	207 (4.2)	33 (8.7)	66 (5.1)	62 (3.2)	46 (3.5)	<0.001
30-day post-discharge	73 (1.4)	16 (4.2)	27 (2.1)	15 (0.7)	15 (1.1)	<0.001
All-cause 30-day readmission	388 (7.8)	49 (12.8)	111 (8.5)	139 (8.0)	89 (6.7)	<0.001
HF	269 (5.4)	42 (11.0)	73 (5.6)	93 (4.7)	61 (4.6)	<0.001
Non-HF	119 (2.4)	7 (1.8)	38 (2.9)	46 (2.3)	28 (2.1)	0.464

STC-related study population	All (4143), *n* (%)	Interval 1 (*n* = 500)	Interval 2 (*n* = 1872)	Interval 3 (*n* = 1771)	*p* Value
All-cause mortality	234 (5.7)	29 (5.8)	122 (6.5)	83 (4.7)	0.057
In-hospital	173 (4.2)	21 (4.2)	90 (4.8)	62 (3.5)	0.143
30-day post-discharge	61 (1.5)	8 (1.6)	32 (1.7)	21 (1.2)	0.410
All-cause 30-day readmission	306 (7.4)	50 (10.0)	141 (7.5)	115 (6.5)	0.150
HF	210 (5.1)	29 (5.8)	99 (5.3)	82 (4.6)	0.484
Non-HF	96 (2.3)	21 (4.2)	42 (2.2)	33 (1.9)	0.009

STC: serum total calcium.

**Figure 2.**
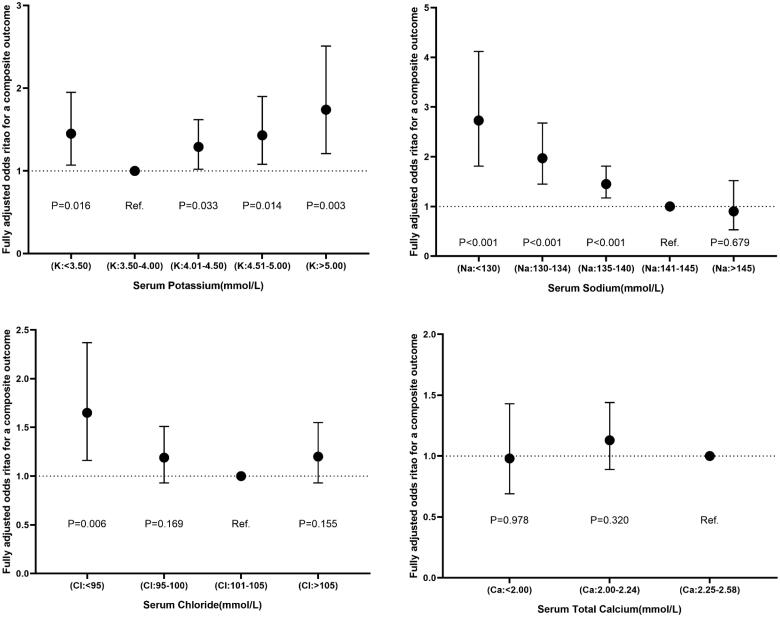
Final adjusted odds ratios with 95% confidence interval of a primary composite outcome in the four serum electrolyte-related study populations.

**Figure 3.**
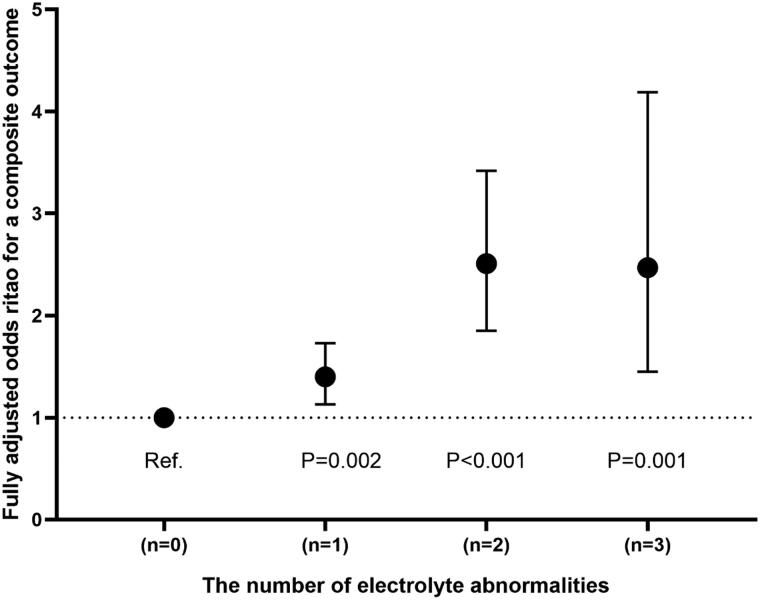
Final adjusted odds ratios with 95% confidence interval comparing a composite outcome among different number of electrolyte abnormalities in the chloride-related study population.

The univariable and multivariable analyses (Table 4 and Supplementary online resource Table 7) showed the OR (95% CI) for the risk of composite outcome according to changes in serum electrolytes when each one was a categorical (intervals) or a continuous variable (median value of each interval). For the potassium-linked study population, patients with a potassium level below 3.5 mmol/L (ORs: 1.45, 95% CI: 1.07–1.95), 4.01–4.50 mmol/L (OR: 1.29, CI: 1.02–1.62), 4.51–5.00 mmol/L (OR: 1.43, CI: 1.08–1.90) and above 5.00 mmol/L (OR: 1.74, CI: 1.21–2.51) had a greater risk of a primary composite outcome as compared to those with a potassium concentrations of 3.50–4.00 mmol/L following full adjustment for potential confounding factors with respect to sex, age, first admission and NYHA or Killip functional class. In contrast, in terms of the sodium-linked study population, compared to participants with sodium at 141–145 mmol/L, sodium levels were inversely related to the risk of a composite outcome after fully adjusting for confounders (sodium < 130 mmol/L: OR, 2.73 (95% CI, 1.81–4.12); 130–134 mmol/L: OR, 1.97 (CI 1.45–2.68); 135–140 mmol/L: OR, 1.45 (CI, 1.17–1.81); and *p* for trend <0.001). However, patients with hypernatremia (>145 mmol/L or interval 5) did not reach a statistical significance of a lower risk of outcome with OR 0.90 (95% CI 0.53–1.52, *p*= 0.679). Notably, we merely found hypochloremia (<95 mmol/L) presenting a higher risk of outcome with an OR of 1.65 (95% CI, 1.16–2.37) in comparison to chloride levels at 101–105 mmol/L. More specifically, participants with serum chloride at 95–100 and >105 mmol/L did not correspond to a significantly increased risk of outcome, with ORs (95% CIs) reaching 1.19 (0.93–1.51) and 1.20 (0.93–1.55), respectively. In addition, the adjusted ORs (95% CI) that compared the STC < 2.00 and 2.00–2.24 vs. 2.25–2.58 mmol/L were 0.98 (0.69–1.43) and 1.13 (0.89–1.44) for a composite outcome, respectively. Furthermore, the number of electrolyte abnormalities was positively related to the risk of a composite outcome (*N* = 1, OR 1.40, 95% CI: 1.13–1.73; *N* = 2, OR 2.51, 95% CI: 1.85–3.42; *N* = 3, OR 2.47, 95% CI: 1.45–4.19; *p* for trend < 0.001) in comparison with *N* = 0. The fully adjusted models were considered well-adjusted in the light of the Hosmer–Lemeshow test (*p*, all > 0.05) and are shown in Figures 2 and 3.

**Table 4.** Odds ratios with the 95% confidence interval for different logistic regression analyses models for a composite outcome (chloride-related study population).

Number of electrolyte abnormalities					
Model	*N* = 0 *n* = 3465	*N* = 1 *n* = 1135	*N* = 2 *n* = 283	*N* = 3 *n* = 83	*p* for trend
Crude model *p* Value	1 (reference)	1.59 (1.29–1.95) <0.001	3.24 (2.41–4.36) <0.001	3.47 (2.09–5.77) <0.001	<0.001
Model 1 *p* Value	1 (reference)	1.57 (1.28–1.94) <0.001	3.21 (2.38–4.32) <0.001	3.54 (2.13–5.89) <0.001	<0.001
Model 2 *p* Value	1 (reference)	1.42 (1.15–1.76) 0.001	2.74 (2.02–3.72) <0.001	2.83 (1.68–4.78) <0.001	<0.001
Model 3 *p* Value	1 (reference)	1.40 (1.13–1.73) 0.002	2.51 (1.85–3.42) <0.001	2.47 (1.45–4.19) 0.001	<0.001

Model 1: adjusted for age and sex. Model 2: further adjusted for first admission, smoking, NYHA or Killip functional class, CAD, AMI, hypertension, VHD, DM, Stroke/TIA, A fib/A flutter and CKD. Model 3: further adjusted for heart rate, SBP, DBP, loop diuretics, ACEI/ARBs, β-blockers, MRA, digitalis, nitrates, nesiritide and statin.

### Subgroup analysis

Clinical profiles (warm or cold) at admission provide important information for ADHF management according to previous studies. We therefore examined the associations between serum electrolyte and the risk of a composite outcome stratified by perfusion status. Similar trends were also observed in the subgroup analysis (Supplementary online resource Table 8).

### Sensitivity analysis

Initially, we used the not imputed or multiple imputed serum albumin, LVEF, eGFR and log (BNP or NT-pro BNP) into model 3 for further adjustment as described above. As expected, all the pooled results were nearly identical to those before the further adjustment in the four study populations. Moreover, similar upshots were observed after excluding the patients with NYHA II. Further information is presented in Table 5 and Supplementary online resource Table 9.

**Table 5.** Sensitivity analyses: logistic regression models of a composite outcome stratified by number of electrolyte abnormalities.

Number of electrolyte abnormalities	A total number of patients (*n* = 4966)				After exclusion of patients with NYHA II (*n* = 4479)			
	*n*	Adjusted^a^ OR (95% CI)	Adjusted^b^ OR (95% CI)	Adjusted^c^ OR (95% CI)	*n*	Adjusted^a^ OR (95% CI)	Adjusted^b^ OR (95% CI)	Adjusted^c^ OR (95% CI)
*N* = 0	3465	1 (reference)	1 (reference)	1 (reference)	3067	1 (reference)	1 (reference)	1 (reference)
*N* = 1	1135	1.40 (1.13–1.73)	1.23 (0.98–1.56)	1.32 (1.07–1.64)	1057	1.47 (1.18–1.83)	1.31 (1.03–1.67)	1.39 (1.12–1.73)
*N* = 2	283	2.51 (1.85–3.42)	2.27 (1.61–3.19)	2.34 (1.71–3.20)	275	2.52 (1.85–3.45)	2.27 (1.60–3.21)	2.35 (1.71–3.22)
*N* = 3	83	2.47 (1.45–4.19)	2.36 (1.32–4.20)	2.24 (1.31–3.82)	80	2.36 (1.37–4.07)	2.41 (1.34–4.33)	2.14 (1.24–3.70)
*p* for trend		<0.001	<0.001	<0.001		<0.001	<0.001	<0.001

OR: odds ratio.

^a^Logistic regression model adjusted for age, sex, first admission, smoking, NYHA or Killip functional class, CAD, AMI, hypertension, VHD, DM, stroke/TIA, A fib/A flutter, CKD, heart rate, SBP, DBP, loop diuretics, ACEI/ARBs, β-blockers, MRA, digitalis, nitrates, nesiritide and statin.

^b^Logistic regression model further adjusted for not imputed LVEF, eGFR, serum albumin and log (BNP or NT-pro BNP).

^c^Logistic regression model further adjusted for multiple imputed LVEF, eGFR, serum albumin and log (BNP or NT-pro BNP).

## Discussion

In this present study, we found significant relationships between three serum electrolytes and poor short-term prognosis in Chinese patients with ADHF. Patients with serum potassium levels at a narrow normal range (3.50–4.00 mmol/L) at admission may herald the lowest risk of recent cardiovascular events. A lower level of baseline serum sodium is associated with a poorer short-term prognosis in patients with ADHF. Notably, serum sodium and chloride levels exhibit discrepancies, in terms of poor short-term prognosis in ADHF. More importantly, this is the first comprehensive evaluation of the number of electrolyte abnormalities among ADHF patients. More specifically, we found that the number of electrolyte abnormalities positively correlates with a poor short-term prognosis of patients with ADHF. Similar trends were also detected in the subgroup analysis.

The relevance of potassium levels to mortality in HF has been studied in previous studies [11,27–29]. Generally speaking, former investigators pinpointed a U-shaped association between the potassium levels and mortality in patients with AHF or other cardiac conditions. On the basis of our study, adjusted ORs for the risk of outcome analogously revealed a U-shaped relevance to potassium levels with a nadir of risk at 3.50–4.00 mmol, the value of which slightly differs from that reported previously [11,30]. Three possible reasons may explain the discrepancies in the findings. First, our study populations involved participants with various diseases, ranging from ADHF springing from AMI (new-onset or not) or decompensation of CHF, whereas previous studies included patients with previous MI or CHF. Second, the baseline serum potassium levels were analysed at the time of admission; therefore, the results were presumably influenced by triggered sympathetic activity which may have led to the transfer of potassium from plasma to the intracellular space during the acute phase among patients with ADHF. Finally, we also counted the 30-day HF readmission as part of the primary outcome as repeated HF hospitalizations not only expend tremendous medical resources but also help in predicting the mortality of patients with HF [31]. Previous studies have indicated that the risk of HF readmission significantly increased in patients with higher concentration of BNP or NT-proBNP, more severe NYHA class, high proportion of rehospitalization for HF, and multiple comorbidities, such as hyperkalaemia (probably discontinuing ACEI/ARB or mineralocorticoid receptor antagonist, MRA), poor renal function or pulmonary infection [32–35]. In our study, participants with potassium (3.50–4.00 mmol/L) possessed lower levels of BNP or NT-proBNP, lesser share of NYHA IV, and rehospitalization for HF, which may partly explain the association of the divergence of serum potassium levels from 3.50 to 4.00 mmol/L with an increased risk of hospitalization for worsening HF. However, our findings did not change after the adjustment of the above-mentioned variables, which implies that the potassium level deviation from 3.50 to 4.00 mmol/L at admission was a risk factor, in terms of the poor recent prognosis in ADHF. In short, the present study with its participants afflicted by all types of ADHF (LVEF ranging from 12% to 82%) expands the previous awareness by displaying an analogous U-shaped relationship between the potassium levels and risk of short-term all-cause mortality or 30-day HF readmission. Furthermore, investigators should pay more attention to the time course of changes in potassium levels during the hospitalization.

Hyponatremia is a common electrolyte abnormity that occurs in hospitalized and non-hospitalized patients with HF. The frequency of hyponatremia was calculated to be approximately 12.7% in our study; a result that is within the range of 7.2–27% observed in early studies due to the distinct populations and subtle discrepancies in reference values [12,25,36,37]. A trove of data have demonstrated that hyponatremia starkly interrelates with an undesirable prognosis of HF, with more adverse events discerned across the escalating hyponatremia severity. Our findings demonstrated that patients with moderate to severe (<130 mmol/L) and mild hyponatremia (130–134 mmol/L) demonstrated a 2.7 times and nearly twofold risk of a composite outcome as compared with sodium levels at 141–145 mmol/L, respectively. Besides, even the population constituting the lower half of the normal sodium range (135–140 mmol/L) exhibited a significant increased risk of outcome (as much as 45%), which is supported by the findings of a former study where the serum sodium levels of 135–139 mmol/L were associated with an elevated mortality risk [38]. However, hypernatremia did not associate with a significant change on the risk of outcome in contrast to that associated with the upper half of the normal sodium range. Apart from the potential reasons we mentioned above, we speculated that the trend could be in part explained by the facts that patients would receive intensive therapies including usage of substantial loop diuretics or salt restriction, which rendered patients with lower levels of normal range of sodium to be potentially hyponatraemic. There might be other explanations for the relationship; however, the findings are intriguing enough to warrant further investigations particularly aiming at ambulatory monitoring of serum sodium both in and out of the hospital setting.

In addition, the increase in risk for outcome was 65% (CI, 16–137%) for participants with hypochloremia vs. with the upper half of the normal chloride range (101–105 mmol/L), which is in line with the results of previous investigations [39,40]. However, the other two groups of patients did not show a significant change in the risk of outcome. This difference was partially caused by the distinct normal reference values, which resulted in the apparent discrepancy in the two study populations (sodium and chloride), in spite of chloride mainly existing as sodium chloride in the extracellular fluid. For example, the number of patients with hyperchloremia was almost five times the patients with hypernatremia; on the contrary, patients within the lower half of the normal range of chloride merely accounted for 55% of their counterparts in the sodium-related study population.

Serum calcium, existing in two forms (bound or ionized) in the body, plays an essential role in myocardial contraction and relaxation [41]. Recently, several investigators have reckoned hypocalcaemia as a portend of poor prognosis in patients with HF. In 2015, Miura et al. indicated that hypocalcaemia is an independent predictor of all-cause mortality in participants living with HF or CKD [42]. Additionally, Jensen et al. demonstrated that altered calcium homeostasis significantly interrelates with a higher 90-day mortality risk in patients with HF [18]. Furthermore, Liu et al. proposed that baseline hypocalcaemia predicts 12-month cardiac rehospitalization and death in patients with HF with preserved ejection fraction (HFpEF) [19]. This phenomenon is arguably due to the fact that extracellular calcium concentration makes an impact on cells with calcium-sensing receptors as well as on excitable cells by influencing their membrane potential [43]. Most of these clinical investigations utilized ionized calcium levels as the measurement. In fact, STC is preferable to ionized calcium in clinical practice across China, as seen in the present study; all of the 13 tertiary hospitals have adopted the monitoring of STC as a routine lab test. Considering that STC is influenced by several factors, we excluded patients with a history of parathyroid disease, vitamin D-related disorders and malignant tumour to minimize the possibility of their effect on the results. Furthermore, extensive potential confounders including CKD, eGFR, serum albumin, serum potassium, serum sodium and physical examinations coupled with medications were considered and used in the multivariable analysis. However, we failed to spot the association of a significant elevated or debased risk of a composite outcome with hypocalcaemia (even the moderate to severe) in contrast to normokalaemia. Therefore, it is imperative to evaluate if the two measurements (ionized calcium and STC) are consistent with each other in predicting the prognosis of specific conditions in further studies.

The association between the number of electrolyte abnormalities and the prognosis of ADHF was seldom discussed in previous studies, in terms of patients with ADHF. In our present study, we found that greater number of electrolyte disorders was associated with a poorer short-term prognosis of ADHF, which may possibly be explained by the fact that patients with greater number of electrolyte disorders usually imply greater severity of HF stage and poorer nutrition status (e.g. higher levels of BNP or NT-proBNP, higher proportions of NYHA IV, and rehospitalizations for HF as well as lower concentrations of serum albumin) [32–35].

Our present study expands previous awareness by displaying different associations between baseline serum electrolyte levels and risk of short-term all-cause mortality or 30-day HF readmission, with several key strengths including prospective data, relatively large study population as well as detailed information on potential confounders,

## Limitations

As this was an observational study, it was impossible to eliminate the residual confounding factors that could bias our results. However, potential confounders were attentively chosen, considering the number of events available, to ensure parsimony of the final models and none of these changed our findings as per the multivariable analyses. Moreover, the serum electrolyte levels were only measured at admission, therefore, time course of changes in ions was not available. Finally, we could barely confirm whether our findings were applicable to other ethnicities.

## Conclusions

In terms of baseline serum electrolyte levels, the deviation of potassium levels from 3.50 to 4.00 mmol/L, lower sodium levels and hypochloremia are associated to poorer short-term prognosis of patients with ADHF. However, there is no association of a higher or lower risk of adverse recent outcome with hypocalcaemia (STC < 2.25 mmol/L). Furthermore, the number of electrolyte abnormalities positively correlated with adverse short-term prognosis of patients with ADHF. These findings imply that electrolyte levels at admission may serve as a useful tool to identify patients with ADHF who are at a high risk of adverse events. Future investigations should fixate the ambulatory monitoring of serum ions and focus on patients with traditionally defined normal serum electrolyte ranges in order to launch more refined reference ranges of serum electrolytes for clinical practice.

## Supplementary Material

Supplemental Material

## Data Availability

The data used to support the findings of this study are available from the corresponding author upon request.
