# Supplementary material for: Associations between serum electrolyte and short-term outcomes in patients with acute decompensated heart failure
Source: Ann Med. 2022 Dec 15;55(1):155–67. doi: 10.1080/07853890.2022.2156595 (PMC9851236; doi:10.1080/07853890.2022.2156595)
Supplement: Supplemental Material [file IANN_A_2156595_SM9464.docx]

**Online Resource Table1.** The univariable analyses of baseline characteristics for a composite outcome

(potassium-related study population)

| Covariate | Level  median (IQR) or n (%) | OR (95%CI) | *P* values |
| --- | --- | --- | --- |
| Age(years) | 69(60-77) | 1.007(1.000-1.014) | 0.049 |
| BMI (kg/m2) | 24.4(22.0-26.8) | 0.953(0.926-0.981) | 0.001 |
| Heart rate(bpm) | 84(71-100) | 1.004(1.001-1.008) | 0.023 |
| SBP (mmHg) | 130(115-147) | 0.990(0.986-0.994) | <0.001 |
| DBP (mmHg) | 80(70-90) | 0.985(0.979-0.991) | <0.001 |
| Serum albumin (g/L) | 38.6(35.4-41.5) | 0.952(0.936-0.970) | <0.001 |
| FBG (mmol/L) | 5.69(4.89-7.28) | 1.044(1.017-1.070) | 0.001 |
| Serum sodium(mmol/L) | 140(137-142) | 0.916(0.900-0.932) | <0.001 |
| eGFR (mL/min/1.73 m2) | 79(59-99) | 0.993(0.990-0.996) | <0.001 |
| LVEF (%) | 45(36-56) | 0.992(0.985-0.999) | 0.019 |
| Sex  Male  Female | 2997(58.3)  2148(41.7) | Reference  0.908(0.760-1.086) | 0.292 |
| Older people  No  Yes | 1885(36.6)  3260(63.4) | Reference  1.034(0.862-1.241) | 0.717 |
| First admission  No  Yes | 2551(49.6)  2594(50.4) | Reference  0.619(0.518-0.740) | <0.001 |
| Smoking  No  Yes | 3917(76.1)  1228(23.9) | Reference  0.773(0.622-0.961) | 0.021 |
| Drinking  No  Yes | 4096(79.6)  1049(20.4) | Reference  0.968(0.778-1.206) | 0.774 |
| NYHA class |  |  | <0.001 |
| Ⅱ | 508(9.9) | Reference |  |
| Ⅲ | 1536(29.9) | 1.461(0.969-2.203) | 0.070 |
| Ⅳ | 2255(43.8) | 2.342(1.587-3.456) | <0.001 |
| Killip class |  |  |  |
| Ⅱ | 563(10.9) | 1.830(1.157-2.894) | 0.01 |
| Ⅲ | 188(3.7) | 3.146(1.846-5.363) | <0.001 |
| Ⅳ | 95(1.8) | 5.690(3.164-10.235) | <0.001 |
| CAD  No  Yes | 2341(45.5)  2804(54.5) | Reference  1.173(0.983-1.401) | 0.077 |
| AMI  No  Yes | 4130(80.3)  1015(19.7) | Reference  1.345(1.094-1.654) | 0.005 |
| Hypertension  No  Yes | 2166(42.1)  2979(57.9) | Reference  0.769(0.645-0.917) | 0.003 |
| VHD  No  Yes | 4390(85.3)  755(14.7) | Reference  1.008(0.788-1.291) | 0.947 |
| DCM  No  Yes | 4424(86.0)  721(14.0) | Reference  1.123(0.880-1.434) | 0.351 |
| DM  No  Yes | 3799(73.8)  1346(26.2) | Reference  1.021(0.837-1.245) | 0.841 |
| Stroke/TIA  No  Yes | 4244(82.5)  901(17.5) | Reference  1.182(0.947-1.474) | 0.139 |
| A fib/A flutter  No  Yes | 3660(71.1)  1485(28.9) | Reference  1.239(1.028-1.495) | 0.025 |
| CKD  No  Yes | 4815(93.6)  330(6.4) | Reference  1.387(1.005-1.913) | 0.047 |
| Loop diuretics  No  Yes | 613(11.9)  4532(88.1) | Reference  1.080(0.819-1.425) | 0.585 |
| Thiazide diuretics  No  Yes | 4677(90.9)  468(9.1) | Reference  0.856(0.621-1.180) | 0.342 |
| ACEI/ARBs  No  Yes | 2378(46.2)  2767(53.8) | Reference  0.662(0.556-0.791) | <0.001 |
| β-blockers  No  Yes | 1612(31.3)  3533(68.7) | Reference  0.828(0.688-0.995) | 0.044 |
| MRA(Spirolactone)  No  Yes | 913(17.7)  4232(82.3) | Reference  0.983(0.782-1.235) | 0.882 |
| Digitalis  No  Yes | 3959(76.9)  1186(23.1) | Reference  1.196(0.978-1.462) | 0.081 |
| Nitrates  No  Yes | 2613(50.8)  2532(49.2) | Reference  1.061(0.891-1.264) | 0.507 |
| Levosimendan  No  Yes | 4903(95.3)  242(4.7) | Reference  1.662(1.170-2.361) | 0.005 |
| Nesiritide  No  Yes | 4693(91.2)  452(8.8) | Reference  1.814(1.396-2.356) | <0.001 |
| Statin  No  Yes | 1704(33.1)  3441(66.9) | Reference  0.818(0.682-0.981) | 0.03 |

BMI body mass index, SBP systolic blood pressure, DBP diastolic blood pressure, FBG fasting blood glucose, eGFR estimated glomerular filtration rate, LVEF left ventricular ejection fraction, NYHA New York Heart Association, CAD coronary artery disease, AMI acute myocardial infarction, VHD valvular heart disease, DCM dilated cardiomyopathy, DM diabetes mellitus, TIA transient ischemic attack, A fib/A flutter atrial fibrillation / atrial flutter, CKD chronic kidney disease, ACEI/ARBs angiotensin-converting enzyme inhibitors/ angiotensin receptor blockers, MRA mineralocorticoid receptor antagonist. Percentages may not total 100 because of rounding.

**Online Resource Table 2.** Distributions of variables with missing data comparing observed complete case data to results from pooling the datasets with imputed variables from multiple imputation.

| **Variables (potassium)** | Level | Number (%)  with missing data | Complete case | Multiple imputation |
| --- | --- | --- | --- | --- |
| eGFR | median (IQR) | 66(1.3) | 79(59-99) | 79(59-99) |
| LVEF (%) | median (IQR) | 124(2.4) | 45(36-56) | 45(36-56) |
| Serum albumin (g/L) | median (IQR) | 165(3.2) | 39(35-42) | 39(35-42) |
| Log(BNP or NT-proBNP) | median (IQR) | 291(5.7) | 3.1(2.7-3.5) | 3.1(2.7-3.5) |
| **Variables (sodium)** | Level | Number (%)  with missing data | Complete case | Multiple imputation |
| eGFR | median (IQR) | 64(1.2) | 79(59-99) | 79(59-99) |
| LVEF (%) | median (IQR) | 124(2.4) | 45(36-56) | 45(36-56) |
| Serum albumin (g/L) | median (IQR) | 164(3.2) | 39(35-42) | 39(35-42) |
| Log(BNP or NT-proBNP) | median (IQR) | 291(5.7) | 3.1(2.7-3.5) | 3.1(2.7-3.5) |
| **Variables (chloride)** | Level | Number (%)  with missing data | Complete case | Multiple imputation |
| eGFR | median (IQR) | 66(1.3) | 79(59-99) | 79(59-99) |
| LVEF (%) | median (IQR) | 121(2.4) | 45(36-56) | 45(36-56) |
| Serum albumin (g/L) | median (IQR) | 159(3.2) | 39(35-42) | 39(35-42) |
| Log(BNP or NT-proBNP) | median (IQR) | 279(5.6) | 3.1(2.7-3.5) | 3.1(2.7-3.5) |
| **Variables (STC)** | Level | Number (%)  with missing data | Complete case | Multiple imputation |
| LVEF (%) | median (IQR) | 104(2.5) | 45(35-57) | 45(35-57) |
| Log(BNP or NT-proBNP) | median (IQR) | 323(7.8) | 3.1(2.7-3.5) | 3.1(2.7-3.5) |

eGFR estimated glomerular filtration rate, LVEF left ventricular ejection fraction, BNP Brain natriuretic peptide, NT-proBNP N-terminal pro brain natriuretic peptide.

**Online Resource Table 3.** Baseline characteristics with regard to five groups of patients classified by serum potassium

| Characteristics | level | interval1  (K: <3.50mmol/L)  (n=631) | interval2  (K:3.50-4.00 mmol/L)  (n=1784) | interval3  (K:4.01-4.50 mmol/L)  (n=1726) | interval4  (K:4.51-5.00 mmol/L)  (n=720) | interval5  (K:>5.00 mmol/L)  (n=284) | *P* values |
| --- | --- | --- | --- | --- | --- | --- | --- |
| Age(years) | median (IQR) | 68(59-77) | 68(60-76) | 69(60-77) | 70(62-78) | 70(62-79) | <0.001 |
| Older people(≥65yr) | n (%) | 387(61.3) | 1101(61.7) | 1098(63.6) | 474(65.8) | 200(70.4) | 0.024 |
| Male | n (%) | 353(55.8) | 1021(57.2) | 1009(58.5) | 452(62.8) | 163(57.4) | 0.074 |
| BMI (kg/m2) | median (IQR) | 24.2(22.0-27.0) | 24.5(22.0-27.0) | 24.5(22.0-27.0) | 24.2(22.0-26.6) | 24.1(21.2-26.1) | 0.123 |
| missing | n(%) | 168(26.6) | 404(22.6) | 419(24.3) | 177(24.6) | 83(29.2) |  |
| First admission | n (%) | 321(50.9) | 984(55.2) | 849(49.2) | 348(48.3) | 92(32.4) | <0.001 |
| Smoking | n (%) | 149(23.6) | 449(25.2) | 395(22.9) | 175(24.3) | 60(21.1) | 0.427 |
| Drinking | n (%) | 127(20.1) | 369(20.7) | 336(19.5) | 168(23.3) | 49(17.3) | 0.159 |
| NYHA class | n (%) |  |  |  |  |  | <0.001 |
| Ⅱ |  | 41(6.5) | 184(10.3) | 197(11.4) | 70(9.7) | 16(5.6) |  |
| Ⅲ |  | 163(25.8) | 514(28.8) | 550(31.9) | 229(31.8) | 80(28.2) |  |
| Ⅳ |  | 325(51.5) | 754(42.3) | 718(41.6) | 314(43.6) | 144(50.7) |  |
| Killip class |  |  |  |  |  |  |  |
| Ⅱ |  | 70(11.1) | 223(12.5) | 174(10.1) | 67(9.3) | 29(10.2) |  |
| Ⅲ |  | 20(3.2) | 77(4.3) | 57(3.3) | 26(3.6) | 8(2.8) |  |
| Ⅳ |  | 12(1.9) | 32(1.8) | 30(1.7) | 14(1.9) | 7(2.5) |  |
| Comorbidity | n (%) |  |  |  |  |  |  |
| CAD |  | 328(52.0) | 948(53.1) | 962(55.7) | 416(57.8) | 150(52.8) | 0.114 |
| AMI |  | 115(18.2) | 389(21.8) | 332(19.2) | 123(17.1) | 56(19.7) | 0.056 |
| Hypertension |  | 376(59.6) | 1042(58.4) | 1001(58.0) | 391(54.3) | 169(59.5) | 0.282 |
| VHD |  | 94(14.9) | 268(15.0) | 262(15.2) | 87(12.1) | 44(15.5) | 0.335 |
| DCM |  | 94(14.9) | 234(13.1) | 250(14.5) | 106(14.7) | 37(13.0) | 0.654 |
| DM |  | 138(21.9) | 417(23.4) | 460(26.7) | 214(29.7) | 117(41.2) | <0.001 |
| Stroke/TIA |  | 107(17.0) | 302(16.9) | 314(18.2) | 118(16.4) | 60(21.1) | 0.366 |
| A fib/ Aflutter |  | 196(31.1) | 528(29.6) | 479(27.8) | 203(28.2) | 79(27.8) | 0.509 |
| CKD |  | 36(5.7) | 76(4.3) | 102(5.9) | 65(9.0) | 51(18.0) | <0.001 |
| PEs | median (IQR) |  |  |  |  |  |  |
| HR (bpm) |  | 86(74-101) | 84(72-100) | 83(70-98) | 83(70-100) | 82(71-100) | 0.015 |
| SBP (mmHg) |  | 131(116-150) | 130(116-147) | 130(116-147) | 128(115-144) | 126(110-147) | 0.011 |
| DBP(mmHg) |  | 80(71-90) | 80(70-90) | 80(70-90) | 78(70-88) | 75(67-88) | <0.001 |
| LVEF(%) |  | 45(35-56) | 45(36-57) | 45(36-57) | 44(35-55) | 43(35-55) | 0.122 |
| missing | n(%) | 13(2.1) | 39(2.2) | 53(3.1) | 15(2.1) | 4(1.4) |  |
| LTs | median (IQR) |  |  |  |  |  |  |
| BNP(pg/ml) |  | 900(427-1732) | 680(293-1409) | 687(312-1442) | 766(343-1572) | 1065(316-2018) | <0.001 |
| missing | n(%) | 229(36.3) | 676(37.9) | 627(36.3) | 286(39.7) | 104(36.6) |  |
| NT-proBNP(ng/ml) |  | 4480(2349-7484) | 3830(1766-6992) | 4504(2214-8993) | 5728(2454-11309) | 6716(2410-15000) | <0.001 |
| missing | n(%) | 420(66.6) | 1185(66.4) | 1149(66.6) | 454(63.1) | 195(68.7) |  |
| eGFR(mL/min/1.73 m2) |  | 82(64-99) | 85(67-103) | 78(59-99) | 71(51-92) | 50(29-74) | <0.001 |
| missing | n(%) | 14(2.2) | 30(1.7) | 12(0.7) | 8(1.1) | 2(0.7) |  |
| FBG(mmol/L) |  | 5.7(4.9-7.0) | 5.7(4.9-7.2) | 5.7(4.8-7.3) | 5.7(4.9-7.5) | 6.2(5.0-8.8) | 0.002 |
| missing | n(%) | 61(9.7) | 142(8.0) | 132(7.6) | 60(8.3) | 26(9.2) |  |
| ALB(g/L) |  | 37(34-41) | 39(36-42) | 39(36-42) | 39(36-42) | 40(36-42) | <0.001 |
| missing | n(%) | 39(6.2) | 67(3.8) | 38(2.2) | 15(2.1) | 6(2.1) |  |
| Sodium(mmol/L) |  | 140(137-143) | 140(138-142) | 140(137-142) | 139(136-141) | 138(134-141) | <0.001 |
| missing | n(%) | 1(0.2) | 6(0.3) | 2(0.1) | 0 | 2(0.7) |  |
| Potassium(mmol/L) |  | 3.32(3.13-3.41) | 3.79(3.66-3.90) | 4.22(4.11-4.35) | 4.70(4.60-4.83) | 5.30(5.11-5.72) | <0.001 |
| BM | n(%) |  |  |  |  |  |  |
| Loop diuretics |  | 564(89.4) | 1551(86.9) | 1507(87.3) | 650(90.3) | 260(91.5) | 0.029 |
| Thiazide diuretics |  | 46(7.3) | 168(9.4) | 165(9.6) | 73(10.1) | 16(5.6) | 0.084 |
| ACEI/ARBs |  | 331(52.5) | 1002(56.2) | 924(53.5) | 382(53.1) | 128(45.1) | 0.01 |
| β-blockers |  | 420(66.6) | 1217(68.2) | 1185(68.7) | 528(73.3) | 183(64.4) | 0.025 |
| MRA(Spirolactone) |  | 538(85.3) | 1471(82.5) | 1403(81.3) | 595(82.6) | 225(79.2) | 0.140 |
| Digitalis |  | 132(20.9) | 396(22.2) | 400(23.2) | 184(25.6) | 74(26.1) | 0.174 |
| Nitrates |  | 303(48) | 856(48) | 862(49.9) | 371(51.5) | 140(49.3) | 0.501 |
| Levosimendan |  | 27(4.3) | 86(4.8) | 89(5.2) | 26(3.6) | 14(4.9) | 0.550 |
| Nesiritide |  | 54(8.6) | 137(7.7) | 156(9.0) | 66(9.2) | 39(13.7) | 0.02 |
| Statin |  | 405(64.2) | 1175(65.9) | 1186(68.7) | 488(67.8) | 187(65.8) | 0.205 |

BMI, body mass index, NYHA New York Heart Association, CAD coronary artery disease, AMI acute myocardial infarction, VHD valvular heart disease, DCM dilated cardiomyopathy, DM diabetes mellitus, TIA transient ischemic attack, A fib/A flutter atrial fibrillation / atrial flutter, CKD chronic kidney disease, PEs physical examinations, HR heart rate, SBP systolic blood pressure, DBP diastolic blood pressure, LVEF left ventricular ejection fraction, LTs laboratory tests, BNP brain natriuretic peptide, NT-pro BNP N-terminal pro brain natriuretic peptide, eGFR estimated glomerular filtration rate, eGFR is calculated using the MDRD eGFR equation as follows: eGFR(mL/min/1.73m2)=186*［serum creatinine (ummol/L)/88.4)］-1.154*(age)-0.203*(0.742 if female), FBG fasting blood glucose, ALB albumin, BM baseline medication, ACEI/ARBs angiotensin-converting enzyme inhibitors/ angiotensin receptor blockers, MRA mineralocorticoid receptor antagonist. Percentages may not total 100 because of rounding.

**Online Resource Table 4.** Baseline characteristics pertaining to five groups of patients categorized by serum sodium

| Characteristics | level | interval1  (Na: <130mmol/L)  (n=170) | interval2  (Na:130-134 mmol/L)  (n=481) | interval3  (Na:135-140 mmol/L)  (n=2378) | interval4  (Na:141-145 mmol/L)  (n=1831) | interval5  (Na: >145mmol/L)  (n=275) | *P* values |
| --- | --- | --- | --- | --- | --- | --- | --- |
| Age(years) | median (IQR) | 75(64-81) | 71(64-79) | 69(61-77) | 67(58-76) | 68(61-76) | <0.001 |
| Older people(≥65yr) | n (%) | 123(72.4) | 354(73.6) | 1530(64.3) | 1074(58.7) | 174(63.3) | <0.001 |
| Male | n (%) | 87(51.2) | 266(55.3) | 1447(60.8) | 1051(57.4) | 142(51.6) | 0.002 |
| BMI (kg/m2) | median (IQR) | 23.7(20.6-26.3) | 23.5(21.3-25.9) | 24.2(22.0-26.7) | 24.6(22.5-27.2) | 24.4(22.2-27.1) | <0.001 |
| missing | n(%) | 49(28.8) | 155(32.2) | 603(25.4) | 375(20.5) | 66(24.0) |  |
| First admission | n (%) | 61(35.9) | 210(43.7) | 1180(49.6) | 979(53.5) | 159(57.8) | <0.001 |
| Smoking | n (%) | 26(15.3) | 85(17.7) | 620(26.1) | 433(23.6) | 61(22.2) | <0.001 |
| Drinking | n (%) | 21(12.4) | 85(17.7) | 510(21.4) | 384(21.0) | 48(17.5) | 0.014 |
| NYHA class | n (%) |  |  |  |  |  | <0.001 |
| Ⅱ |  | 7(4.1) | 27(5.6) | 224(9.4) | 215(11.7) | 34(12.4) |  |
| Ⅲ |  | 34(20.0) | 100(20.8) | 669(28.1) | 632(34.5) | 102(37.1) |  |
| Ⅳ |  | 105(60.8) | 248(51.6) | 1025(43.1) | 759(41.5) | 112(40.7) |  |
| Killip class |  |  |  |  |  |  |  |
| Ⅱ |  | 12(7.1) | 71(14.8) | 306(12.9) | 151(8.2) | 21(7.6) |  |
| Ⅲ |  | 8(4.7) | 22(4.6) | 107(4.5) | 46(2.5) | 4(1.5) |  |
| Ⅳ |  | 4(2.4) | 13(2.7) | 47(2.0) | 28(1.5) | 2(0.7) |  |
| Comorbidity | n (%) |  |  |  |  |  |  |
| CAD |  | 83(48.8) | 302(62.8) | 1357(57.1) | 910(49.7) | 144(52.4) | <0.001 |
| AMI |  | 27(15.9) | 137(28.5) | 535(22.5) | 275(15.0) | 37(13.5) | <0.001 |
| Hypertension |  | 83(48.8) | 262(54.5) | 1328(55.8) | 1125(61.4) | 178(64.7) | <0.001 |
| VHD |  | 33(19.4) | 77(16.0) | 335(14.1) | 270(14.7) | 40(14.5) | 0.357 |
| DCM |  | 28(16.5) | 60(12.5) | 328(13.8) | 264(14.4) | 39(14.2) | 0.702 |
| DM |  | 42(24.7) | 167(34.7) | 654(27.5) | 416(22.7) | 67(24.4) | <0.001 |
| Stroke/TIA |  | 24(14.1) | 95(19.8) | 437(18.4) | 294(16.1) | 51(18.5) | 0.122 |
| A fib/ A flutter |  | 54(31.8) | 143(29.7) | 662(27.8) | 552(30.1) | 71(25.8) | 0.352 |
| CKD |  | 14(8.2) | 43(8.9) | 156(6.6) | 95(5.2) | 22(8.0) | 0.017 |
| PEs | median (IQR) |  |  |  |  |  |  |
| HR (bpm) |  | 82(68-98) | 84(72-100) | 84(72-99) | 84(70-100) | 84(71-100) | 0.582 |
| SBP (mmHg) |  | 126(108-142) | 122(109-139) | 129(114-146) | 132(120-150) | 136(122-152) | <0.001 |
| DBP(mmHg) |  | 72(65-85) | 75(66-85) | 79(70-89) | 80(71-91) | 81(72-94) | <0.001 |
| LVEF(%) |  | 45(33-55) | 43(35-56) | 45(36-56) | 45(37-57) | 46(34-57) | 0.021 |
| missing | n(%) | 6(3.5) | 15(3.1) | 68(2.9) | 30(1.6) | 5(1.8) |  |
| LTs | median (IQR) |  |  |  |  |  |  |
| BNP(pg/ml) |  | 1540(660-2580) | 928(427-1835) | 760(300-1573) | 670(309-1370) | 776(380-1475) | <0.001 |
| missing | n(%) | 87(51.2) | 213(44.3) | 988(41.5) | 568(31.0) | 63(22.9) |  |
| NT-proBNP(ng/ml) |  | 7507(2730-13052) | 6216(2727-12407) | 4350(1966-8565) | 3820(1960-7069) | 4840(2960-9902) | <0.001 |
| missing | n(%) | 92(54.1) | 282(58.6) | 1497(63.0) | 1305(71.3) | 220(80.0) |  |
| eGFR(mL/min/1.73 m2) |  | 68(41-92) | 71(50-92) | 78(58-100) | 82(64-101) | 81(59-98) | <0.001 |
| missing | n(%) | 3(1.8) | 8(1.7) | 34(1.4) | 16(0.9) | 3(1.1) |  |
| FBG(mmol/L) |  | 6.1(5.0-8.7) | 6.6(5.3-9.4) | 5.9(5.0-7.6) | 5.4(4.7-6.6) | 5.3(4.7-6.2) | <0.001 |
| missing | n(%) | 25(14.7) | 41(8.5) | 198(8.3) | 136(7.4) | 19(6.9) |  |
| ALB(g/L) |  | 37(34-40) | 37(34-40) | 39(35-42) | 39(36-42) | 39(36-41) | <0.001 |
| missing | n(%) | 11(6.5) | 41(8.5) | 83(3.5) | 45(2.5) | 8(2.9) |  |
| Potassium (mmol/L) |  | 4.3(3.9-4.9) | 4.2(3.8-4.6) | 4.1(3.8-4.4) | 4.0(3.7-4.3) | 3.9(3.6-4.2) | <0.001 |
| Sodium (mmol/L) |  | 127(124-129) | 133(132-134) | 138(137-139) | 142(141-143) | 146(146-147) | <0.001 |
| BM | n(%) |  |  |  |  |  |  |
| Loop diuretics |  | 154(90.6) | 438(91.1) | 2087(87.8) | 1605(87.7) | 242(88.0) | 0.228 |
| Thiazide diuretics |  | 16(9.4) | 51(10.6) | 222(9.3) | 165(9.0) | 14(5.1) | 0.144 |
| ACEI/ARBs |  | 89(52.4) | 241(50.1) | 1233(51.9) | 1043(57.0) | 156(56.7) | 0.005 |
| β-blockers |  | 103(60.6) | 305(63.4) | 1654(69.6) | 1283(70.1) | 181(65.8) | 0.005 |
| MRA(Spirolactone) |  | 143(84.1) | 403(83.8) | 1949(82.0) | 1502(82.0) | 228(82.9) | 0.135 |
| Digitalis |  | 47(27.6) | 123(25.6) | 540(22.7) | 423(23.1) | 52(18.9) | 0.161 |
| Nitrates |  | 66(38.8) | 262(54.5) | 1179(49.6) | 883(48.2) | 137(49.8) | 0.009 |
| Levosimendan |  | 12(7.1) | 24(5.0) | 117(4.9) | 75(4.1) | 13(4.7) | 0.412 |
| Nesiritide |  | 24(14.1) | 67(13.9) | 209(8.8) | 133(7.3) | 17(6.2) | <0.001 |
| Statin |  | 95(55.9) | 323(67.2) | 1605(67.5) | 1220(66.6) | 190(69.1) | 0.035 |

BMI, body mass index, NYHA New York Heart Association, CAD coronary artery disease, AMI acute myocardial infarction, VHD valvular heart disease, DCM dilated cardiomyopathy, DM diabetes mellitus, TIA transient ischemic attack, A fib/A flutter atrial fibrillation / atrial flutter, CKD chronic kidney disease, PEs physical examinations, HR heart rate, SBP systolic blood pressure, DBP diastolic blood pressure, LVEF left ventricular ejection fraction, LTs laboratory tests, BNP brain natriuretic peptide, NT-pro BNP N-terminal pro brain natriuretic peptide, eGFR estimated glomerular filtration rate, FBG fasting blood glucose, ALB albumin, BM baseline medication, ACEI/ARBs angiotensin-converting enzyme inhibitors/ angiotensin receptor blockers, MRA mineralocorticoid receptor antagonist. Percentages may not total 100 because of rounding.

**Online Resource Table 5.** Baseline characteristics concerning four groups of participants assorted by serum chloride.

| Characteristics | level | interval1  (Cl: <95mmol/L)  (n=381) | interval2  (Cl: 95-100mmol/L)  (n=1297) | interval3  (Cl: 101-105mmol/L)  (n=1964) | interval4  (Cl: >105mmol/L)  (n=1324) | *P* values |
| --- | --- | --- | --- | --- | --- | --- |
| Age(years)  Older people(≥65yr) | median (IQR)  n (%) | 71(62-79)  258(67.7) | 69(61-78)  851(65.6) | 68(60-77)  1239(63.1) | 68(60-76)  810(61.2) | <0.001  0.035 |
| Male | n (%) | 194(50.9) | 766(59.1) | 1162(59.2) | 758(57.3) | 0.02 |
| BMI (kg/m2)  missing | median (IQR)  n(%) | 23.8(20.8-26.1)  128(33.6) | 24.1(21.6-26.3)  354(27.4) | 24.5(22.3-27.3)  453(23.1) | 24.5(22.2-26.9)  297(22.4) | <0.001 |
| First admission | n (%) | 130(34.1) | 598(46.1) | 1044(53.2) | 735(55.5) | <0.001 |
| Smoking | n (%) | 63(16.5) | 281(21.7) | 492(25.1) | 358(27.0) | <0.001 |
| Drinking | n (%) | 58(15.2) | 236(18.2) | 437(22.3) | 292(22.1) | <0.001 |
| NYHA class | n (%) |  |  |  |  | <0.001 |
| Ⅱ |  | 14(3.7) | 97(7.5) | 218(11.1) | 158(11.9) |  |
| Ⅲ |  | 100(26.2) | 344(26.5) | 618(31.5) | 420(31.7) |  |
| Ⅳ |  | 220(57.7) | 602(46.4) | 832(42.4) | 530(40.0) |  |
| Killip class |  |  |  |  |  |  |
| Ⅱ |  | 31(8.1) | 158(12.2) | 207(10.5) | 147(11.1) |  |
| Ⅲ |  | 12(3.1) | 64(4.9) | 61(3.1) | 41(3.1) |  |
| Ⅳ |  | 4(1.0) | 32(2.5) | 28(1.4) | 28(2.1) |  |
| Comorbidity  CAD  AMI  Hypertension  VHD  DCM  DM  Stroke/TIA  A fib/A flutter  CKD | n (%) | 200(52.5)  62(16.3)  180(47.2)  76(19.9)  60(15.7)  98(25.7)  68(17.8)  129(33.9)  31(8.1) | 742(57.2)  302(23.3)  744(57.4)  185(14.3)  184(14.2)  393(30.3)  237(18.3)  394(30.4)  86(6.6) | 1055(53.7)  363(18.5)  1160(59.1)  274(14.0)  278(14.2)  514(26.2)  346(17.6)  555(28.3)  119(6.1) | 715(54.0)  252(19.0)  801(60.5)  193(14.6)  165(12.5)  300(22.7)  228(17.2)  357(27.0)  82(6.2) | 0.168  0.001  <0.001  0.024  0.312  <0.001  0.915  0.033  0.474 |
| Physical examinations  Heart rate(bpm)  SBP (mmHg)  DBP (mmHg)  LVEF (%)  missing | median (IQR)  n(%) | 86(70-103)  120(108-137)  73(65-85)  44(33-57)  14(3.7) | 86(73-101)  127(112-145)  79(70-89)  44(35-56)  39(3.0) | 84(72-99)  130(117-147)  80(70-90)  45(36-56)  45(2.3) | 82(70-98)  134(120-150)  80(70-91)  46(37-58)  23(1.7) | <0.001  <0.001  <0.001  <0.001 |
| Laboratory tests  BNP (pg/ml)  missing  NT-proBNP (pg/ml)  missing  eGFR (mL/min/1.73 m2)  missing  FBG (mmol/L)  missing  Serum albumin (g/L)  missing  potassium(mmol/L)  sodium(mmol/L)  chloride(mmol/L) | median (IQR)  n(%)  n(%)  n(%)  n(%)  n(%) | 1065(461-2150)  153(40.2)  6866(2442-13492)  241(63.3)  69(47-94)  7(1.8)  6.0(5.1-8.4)  42(11.))  38(34-41)  18(4.7)  4.0(3.6-4.5)  133(129-136)  92(89-94) | 852(325-1724)  512(39.5)  4790(2295-9311)  820(63.2)  77(57-97)  23(1.8)  6.1(5.0-8.2)  132(10.2)  39(36-42)  50(3.9)  4.1(3.7-4.4)  138(135-140)  99(97-100) | 643(302-1320)  749(38.1)  4090(1868-7991)  1292(65.8)  81(61-99)  18(0.9)  5.6(4.8-7.1)  160(8.1)  39(36-42)  61(3.1)  4.0(3.7-4.4)  140(138-142)  103(102-104) | 708(309-1446)  422(31.9)  3902(1930-7150)  947(71.5)  82(63-102)  18(1.4)  5.5(4.9-6.7)  73(5.5)  38(35-41)  30(2.3)  4.1(3.8-4.4)  142(140-144)  107(106-109) | <0.001  <0.001  <0.001  <0.001  <0.001  0.363  <0.001  <0.001 |
| Baseline medications  Loop diuretics  Thiazide diuretics  ACEI/ARBs  β-blockers  MRA(Spirolactone)  Digitalis  Nitrates  Levosimendan  Nesiritide  Statin | n (%) | 350(91.9)  34(8.9)  170(44.6)  245(64.3)  324(85.0)  117(30.7)  164(43.0)  26(6.8)  41(10.8)  206(54.1) | 1186(91.4)  109(8.4)  653(50.3)  874(67.4)  1117(86.1)  325(25.1)  625(48.2)  57(4.4)  120(9.3)  854(65.8) | 1721(87.6)  177(9.0)  1109(56.5)  1351(68.8)  1618(82.4)  421(21.4)  977(49.7)  74(3.8)  154(7.8)  1346(68.5) | 1109(83.8)  131(9.9)  706(53.3)  912(68.9)  1034(78.1)  303(22.9)  703(53.1)  70(5.3)  107(8.1)  932(70.4) | <0.001  0.614  <0.001  0.304  <0.001  0.001  0.003  0.029  0.184  <0.001 |

BMI, body mass index, NYHA New York Heart Association, CAD coronary artery disease, AMI acute myocardial infarction, VHD valvular heart disease, DCM dilated cardiomyopathy, DM diabetes mellitus, TIA transient ischemic attack, A fib/A flutter atrial fibrillation / atrial flutter, CKD chronic kidney disease, SBP systolic blood pressure, DBP diastolic blood pressure, LVEF left ventricular ejection fraction, BNP brain natriuretic peptide, NT-pro BNP N-terminal pro brain natriuretic peptide, eGFR estimated glomerular filtration rate, FBG fasting blood glucose, ACEI/ARBs angiotensin-converting enzyme inhibitors/ angiotensin receptor blockers, MRA mineralocorticoid receptor antagonist. Percentages may not total 100 because of rounding.

**Online Resource Table 6.** Baseline characteristics regarding three groups of patients stratified by STC.

| Characteristics | level | interval1  (Ca: <2.00mmol/L)  (n=500) | interval2  (Ca: 2.00-2.24mmol/L)  (n=1872) | interval3  (Ca: 2.25-2.58mmol/L)  (n=1771) | *P* values |
| --- | --- | --- | --- | --- | --- |
| Age(years)  Older people(≥65yr) | median (IQR)  n (%) | 69(61-78)  324(64.8) | 69(61-78)  1242(66.3) | 67(59-65)  1044(58.9) | <0.001  <0.001 |
| Male | n (%) | 267(53.4) | 1149(61.4) | 1015(57.3) | 0.002 |
| BMI (kg/m2)  missing | median (IQR)  n(%) | 24.2(21.7-27.0)  93(18.6) | 24.2(21.9-26.7)  410(21.9) | 24.5(22.2-27.0)  366(20.7) | 0.047 |
| First admission | n (%) | 244(48.8) | 1004(53.6) | 864(48.8) | 0.008 |
| Smoking | n (%) | 125(25.0) | 469(25.1) | 385(21.7) | 0.047 |
| Drinking | n (%) | 105(21.0) | 374(20.0) | 332(18.7) | 0.447 |
| NYHA class | n (%) |  |  |  | <0.001 |
| Ⅱ |  | 47(9.4) | 138(7.4) | 218(12.3) |  |
| Ⅲ |  | 114(22.8) | 544(29.1) | 608(34.3) |  |
| Ⅳ |  | 247(49.4) | 845(45.1) | 717(40.5) |  |
| Killip class |  |  |  |  |  |
| Ⅱ |  | 53(10.6) | 224(12.0) | 160(9.0) |  |
| Ⅲ |  | 23(4.6) | 81(4.3) | 46(2.6) |  |
| Ⅳ |  | 16(3.2) | 40(2.1) | 22(1.2) |  |
| Comorbidity  CAD  AMI  Hypertension  VHD  DCM  DM  Stroke/TIA  A fib/A flutter  CKD | n (%) | 295(59.0)  92(18.4)  312(62.4)  72(14.4)  64(12.8)  133(26.6)  93(18.6)  140(28.0)  50(10.0) | 1088(58.1)  345(18.4)  1060 (56.6)  265(14.2)  233(12.4)  455(24.3)  331(17.7)  478(25.5)  129(6.9) | 951(53.7)  228(12.9)  1009(57.0)  270(15.2)  286(16.1)  464(26.2)  279(15.8)  551(31.1)  89(5.0) | 0.012  <0.001  0.058  0.640  0.004  0.339  0.176  0.001  <0.001 |
| Physical examinations  Heart rate(bpm)  SBP (mmHg)  DBP (mmHg)  LVEF (%)  missing | median (IQR)  n(%) | 86(74-102)  130(115-151)  80(70-90)  44(33-55)  10(2.0) | 83(70-99)  130(115-148)  78(70-89)  46(36-58)  50(2.7) | 83(70-100)  129(115-145)  80(70-90)  44(35-58)  44(2.5) | 0.003  0.094  0.01  <0.001 |
| Laboratory tests  BNP (pg/ml)  missing  NT-proBNP (pg/ml)  missing  eGFR (mL/min/1.73 m2)  FBG (mmol/L)  missing  albumin (g/L)  potassium(mmol/L)  sodium(mmol/L)  STC (mmol/L) | median (IQR)  n(%)  n(%)  n(%) | 913(441-1766)  172(34.4)  4312(1930-8780)  357(71.4)  77(56-97)  6.0(4.9-9.8)  34(6.8)  35(32-39)  4.0(3.7-4.4)  140(136-142)  1.24(1.17-1.87) | 882(400-1680)  834(44.6)  4847(2419-9143)  1114(59.5)  80(59-101)  5.7(4.8-7.3)  135(7.2)  37(34-39)  4.0(3.6-4.3)  139(136-142)  2.16(2.10-2.20) | 648(282-1443)  750(42.3)  3896(1950-7612)  1137(64.2)  79(60-98)  5.7(5.0-7.3)  133(7.5)  41(38-43)  4.1(3.8-4.5)  140(138-142)  2.33(2.28-2.39) | <0.001  0.004  0.148  0.135  <0.001  <0.001  <0.001  <0.001 |
| Baseline medications  Loop diuretics  Thiazide diuretics  ACEI/ARBs  β-blockers  MRA(Spirolactone)  Digitalis  Nitrates  Levosimendan  Nesiritide  Statin | n (%) | 452(90.4)  63(12.6)  250(50.0)  301(60.2)  331(66.2)  94(18.8)  357(71.4)  11 (2.2)  21(4.2)  347(69.4) | 1673(89.4)  136(7.3)  1026(54.8)  1261(67.4)  1573(84.0)  401(21.4)  1090(58.2)  109(5.8)  189(10.1)  1217(65.0) | 1559 (88.0)  132(7.5)  964(54.4)  1269(71.7)  1488(84.0)  392(22.1)  961(54.3)  108(6.1)  119(6.7)  1121(63.3) | 0.232  <0.001  0.147  <0.001  <0.001  0.276  <0.001  0.002  <0.001  0.04 |

STC serum total calcium, BMI body mass index, NYHA New York Heart Association, CAD coronary artery disease, AMI acute myocardial infarction, VHD valvular heart disease, DCM dilated cardiomyopathy, DM diabetes mellitus, TIA transient ischemic attack, A fib/A flutter atrial fibrillation / atrial flutter, CKD chronic kidney disease, SBP systolic blood pressure, DBP diastolic blood pressure, LVEF left ventricular ejection fraction, BNP brain natriuretic peptide, NT-pro BNP N-terminal pro brain natriuretic peptide, eGFR estimated glomerular filtration rate, FBG fasting blood glucose, ACEI/ARBs angiotensin-converting enzyme inhibitors/ angiotensin receptor blockers, MRA mineralocorticoid receptor antagonist. Percentages may not total 100 because of rounding.

**Online Resource Table 7** Odds Ratios with the 95% Confidence interval for different logistic Regression Analyses models for a composite outcome. (Potassium, Sodium, Chloride and STC related study populations )

| Serum electrolytes (mmol/L) |  | Model | | | |
| --- | --- | --- | --- | --- | --- |
| Serum potassium | n(5145) | Crude model  *P* values | Model1  *P* values | Model2  *P* values | Model3  *P* values |
| interval1 (<3.50) | 631 | 1.51(1.13-2.03)  0.005 | 1.51(1.13-2.03)  0.005 | 1.45(1.08-1.95)  0.013 | 1.45(1.07-1.95)  0.016 |
| interval2 (3.50-4.00) | 1784 | 1(reference) | 1(reference) | 1(reference) | 1(reference) |
| interval3(4.01-4.50) | 1726 | 1.34(1.07-1.68)  0.011 | 1.33(1.06-1.67)  0.011 | 1.33(1.06-1.68)  0.013 | 1.29(1.02-1.62)  0.033 |
| interval4(4.51-5.00) | 720 | 1.62(1.23-2.13)  0.001 | 1.59(1.20-2.09)  0.001 | 1.57(1.19-2.08)  0.002 | 1.43(1.08-1.90)  0.014 |
| interval5(>5.00) | 284 | 2.50(1.78-3.52)  <0.001 | 2.46(1.74-3.46)  <0.001 | 2.15(1.50-3.06)  <0.001 | 1.74(1.21-2.51)  0.003 |
| *P* for trend |  | - | - | - | 0.043 |
| Serum sodium | n(5135) |  |  |  |  |
| interval1 (<130) | 170 | 3.96(2.68-5.87)  <0.001 | 3.91(2.64-5.80)  <0.001 | 3.21(2.15-4.79)  <0.001 | 2.73 (1.81-4.12)  <0.001 |
| interval2 (130-134) | 481 | 2.71(2.03-3.63)  <0.001 | 2.67(2.03-3.63)  <0.001 | 2.20(1.63-2.97)  <0.001 | 1.97(1.45-2.68)  <0.001 |
| interval3 (135-140) | 2378 | 1.67(1.35-2.07)  <0.001 | 1.67(1.33-2.05)  <0.001 | 1.53(1.23-1.90)  <0.001 | 1.45(1.17-1.81)  <0.001 |
| interval4(141-145) | 1831 | 1(reference) | 1(reference) | 1(reference) | 1(reference) |
| interval5(>145)) | 275 | 0.82(0.49-1.38)  0.459 | 0.82(0.49-1.39)  0.466 | 0.87(0.51-1.47)  0.601 | 0.90(0.53-1.52)  0.679 |
| *P* for trend |  | - | - | - | <0.001 |
| Serum chloride | n(4966) |  |  |  |  |
| interval1 (<95) | 381 | 3.31(2.50-4.40)  <0.001 | 3.31(2.49-4.40)  <0.001 | 2.83(2.12-3.79)  <0.001 | 1.65(1.16-2.37)  0.006 |
| interval2 (95-100) | 1297 | 1.55(1.24-1.94)  <0.001 | 1.54(1.23-1.93)  <0.001 | 1.39(1.10-1.75)  0.005 | 1.19(0.93-1.51)  0.169 |
| interval3(101-105) | 1964 | 1(reference) | 1(reference) | 1(reference) | 1(reference) |
| interval4(>105) | 1324 | 1.07(0.84-1.37)  0.581 | 1.08(0.84-1.37)  0.555 | 1.10(0.86-1.41)  0.459 | 1.20(0.93-1.55)  0.155 |
| *P* for trend |  | - | - | - | 0.125 |
| Serum total calcium | n(4143) |  |  |  |  |
| interval1 (<2.00) | 500 | 1.28(0.93-1.75)  0.131 | 1.26(0.92-1.74)  0.151 | 1.10(0.86-1.41)  0.459 | 0.99(0.69-1.43)  0.978 |
| interval2 (2.00-2.24) | 1872 | 1.30(1.05-1.61)  0.015 | 1.27(1.03-1.57)  0.029 | 1.18(0.85-1.63)  0.318 | 1.14(0.89-1.45)  0.320 |
| interval3 (2.25-2.58) | 1771 | 1(reference) | 1(reference) | 1(reference) | 1(reference) |
| *P* for trend |  | - | - | - | 0.742 |

**Potassium-related study population**

Model1: adjusted for age and sex.

Model2: further adjusted for first admission, smoking, NYHA or Killip functional class, CAD, AMI, hypertension, DM, Stroke/TIA, A fib/A flutter and CKD.

Model3: further adjusted for heart rate, SBP, DBP, serum sodium, Loop diuretics, ACEI/ARBs, β-blockers, Digitalis, Levosimendan, Nesiritide and Statin.

**Sodium-related study population**

Model1: adjusted for age and sex.

Model2: further adjusted for first admission, smoking, drinking, NYHA or Killip functional class, CAD, AMI, hypertension, DM, Stroke/TIA, A fib/A flutter, and CKD.

Model3: further adjusted for heart rate, SBP, DBP, serum potassium, Loop diuretics, ACEI/ARBs, β-blockers, Digitalis, Levosimendan, Nesiritide, and Statin.

**Chloride-related study population**

Model1: adjusted for age and sex.

Model2: further adjusted for first admission, smoking, drinking, NYHA or Killip functional class, CAD, AMI, hypertension, VHD, DM, Stroke/TIA, A fib/A flutter, and CKD.

Model3: further adjusted for heart rate, SBP, DBP, serum potassium, serum sodium, Loop diuretics, ACEI/ARBs, β-blockers, MRA, Digitalis, Nitrates, Levosimendan, Nesiritide, and Statin.

**STC-related study population**

Model1: adjusted for age and sex.

Model2: further adjusted for first admission, smoking, NYHA or Killip functional class, CAD, AMI, hypertension, DCM, Stroke/TIA, A fib/A flutter, and CKD.

Model3: further adjusted for heart rate, SBP, DBP, eGFR, serum albumin ,serum potassium, serum sodium, Thiazide diuretics, ACEI/ARBs, β-blockers, MRA, Nitrates, Levosimendan, Nesiritide, and Statin.

**Online Resource Table 8.** Odds Ratios with the 95% Confidence Interval for distinct logistic Regression Analyses models for a composite outcome, by perfusion status.

| Variable | Adequate perfusion | | Compromised perfusion | | *P* for interaction |
| --- | --- | --- | --- | --- | --- |
| Potassium(mmol/L) | n | Adjusted^a^ OR(95%CI) | n | Adjusted^a^ OR(95%CI) | 0.447 |
| interval1(<3.50) | 546 | 1.27(0.91 -1.78) | 85 | 2.34(1.15-4.76) |  |
| interval2(3.50-4.00) | 1586 | 1(reference) | 198 | 1(reference) |  |
| Interval3(4.01-4.50) | 1530 | 1.25(0.98 -1.61) | 196 | 1.55(0.84-2.87) |  |
| Interval4(4.51-5.00) | 646 | 1.42(1.04 -1.93) | 74 | 1.66(0.75-3.70) |  |
| Interval5(>5.00) | 248 | 1.58(1.06-2.36) | 36 | 2.68(1.07-6.66) |  |
| *P* for trend |  | 0.045 |  | 0.500 |  |
| Sodium (mmol/L) | n | Adjusted^b^ OR(95%CI) | n | Adjusted^b^ OR(95%CI) |  |
| interval1 (<130) | 149 | 3.03(1.94-4.74) | 21 | 2.79(0.86-9.08) | 0.258 |
| Interval2 (130-134) | 404 | 2.31(1.65-3.22) | 77 | 1.07(0.49-2.34) |  |
| Interval3 (135-140) | 2109 | 1.54(1.21-1.96) | 269 | 1.36(0.77-2.42) |  |
| Interval4 (141-145) | 1636 | 1(reference) | 195 | 1(reference) |  |
| Interval5 (>145) | 251 | 0.65(0.34-1.22) | 24 | 2.70(0.85-8.57) |  |
| *P* for trend |  | <0.001 |  | 0.612 |  |
| Chloride(mmol/L) | n | Adjusted^c^ OR(95%CI) | n | Adjusted^c^ OR(95%CI) | 0.442 |
| interval1 (<95) | 321 | 1.73(1.17-2.57) | 60 | 1.31(0.54-3.21) |  |
| Interval2 (95-100) | 1123 | 1.18(0.90-1.54) | 174 | 1.42(0.77-2.62) |  |
| Interval3 (101-105) | 1756 | 1(reference) | 208 | 1(reference) |  |
| Interval4 (>105) | 1195 | 1.21(0.91-1.59) | 129 | 1.33(0.66-2.67) |  |
| *P* for trend |  | 0.115 |  | 0.723 |  |
| Number of electrolyte abnormalities | n | Adjusted^d^ OR(95%CI) | n | Adjusted^d^ OR(95%CI) | 0.122 |
| N=0 | 3110 | 1(reference) | 355 | 1(reference) |  |
| N=1 | 973 | 1.28(1.01-1.62) | 162 | 1.91(1.14-3.21) |  |
| N=2 | 244 | 2.69(1.93-3.75) | 39 | 1.60(0.68-3.77) |  |
| N=3 | 68 | 2.76(1.55-4.91) | 15 | 1.17(0.27-4.99) |  |
| *P* for trend |  | <0.001 |  | 0.098 | |

OR odds ratio

^a^Logistic regression model adjusted for age, sex, first admission, smoking, NYHA or Killip functional class, CAD, AMI, hypertension, DM, Stroke/TIA, A fib/A flutter, CKD. heart rate, SBP, DBP, serum sodium, Loop diuretics, ACEI/ARBs, β-blockers, Digitalis, Levosimendan, Nesiritide and Statin.

^b^Logistic regression model adjusted for age, sex, first admission, smoking, drinking, NYHA or Killip functional class, CAD, AMI, hypertension, DM, Stroke/TIA, A fib/A flutter, CKD, heart rate, SBP, DBP, serum potassium, Loop diuretics, ACEI/ARBs, β-blockers, Digitalis, Levosimendan, Nesiritide, and Statin.

^c^Logistic regression model adjusted for age, sex, first admission, smoking, drinking, NYHA or Killip functional class, CAD, AMI, hypertension, VHD, DM, Stroke/TIA, A fib/A flutter ,CKD, heart rate, SBP, DBP, serum potassium, serum sodium, Loop diuretics, ACEI/ARBs, β-blockers, MRA, Digitalis, Nitrates, Levosimendan, Nesiritide and Statin.

^d^Logistic regression model adjusted for age, sex, first admission, smoking, NYHA or Killip functional class, CAD, AMI, hypertension, VHD, DM, Stroke/TIA, A fib/A flutter, CKD, heart rate, SBP, DBP, Loop diuretics, ACEI/ARBs, β-blockers, MRA, Digitalis, Nitrates, Nesiritide and, Statin.

**Online Resource Table 9.** Sensitivity analyses: logistic regression models of a composite outcome among different groups of serum electrolytes

| Serum electrolytes (mmol/L) |  | A total number of patients | | |  | After exclusion of patients with NYHAⅡ | | |
| --- | --- | --- | --- | --- | --- | --- | --- | --- |
| Serum potassium | n(5145) | Adjusted^a^ OR  (95%CI) | Adjusted^b^ OR  (95%CI) | Adjusted^c^ OR  (95%CI) | (n=4637) | Adjusted^a^ OR  (95%CI) | Adjusted^b^ OR  (95%CI) | Adjusted^c^ OR  (95%CI) |
| interval1 (<3.50) | 631 | 1.45(1.07-1.95) | 1.28(0.91-1.78) | 1.42(1.05-1.91) | 590 | 1.48(1.09-2.00) | 1.35(0.96-1.89) | 1.44(1.06-1.96) |
| interval2 (3.50-4.00) | 1784 | 1(reference) | 1(reference) | 1(reference) | 1600 | 1(reference) | 1(reference) | 1(reference) |
| interval3(4.01-4.50) | 1726 | 1.29(1.02-1.62) | 1.21(0.94-1.56) | 1.27(1.01-1.60) | 1529 | 1.29(1.01-1.64) | 1.21(0.93-1.57) | 1.27(1.00-1.62) |
| interval4(4.51-5.00) | 720 | 1.43(1.08-1.90) | 1.42(1.05-1.93) | 1.39(1.04-1.85) | 650 | 1.51(1.12-2.02) | 1.49(1.09-2.04) | 1.46(1.08-1.97) |
| interval5(>5.00) | 284 | 1.74(1.21-2.51) | 1.60(1.07-2.39) | 1.67(1.15-2.42) | 268 | 1.79(1.23-2.59) | 1.67(1.11-2.51) | 1.71(1.17-2.49) |
| *P* for trend |  | 0.043 | 0.05 | 0.082 |  | 0.032 | 0.046 | 0.063 |
| Serum sodium | n(5135) | Adjusted^a^ OR  (95%CI) | Adjusted^b^ OR  (95%CI) | Adjusted^c^ OR  (95%CI) | n(4628) | Adjusted^a^ OR  (95%CI) | Adjusted^b^ OR  (95%CI) | Adjusted^c^ OR  (95%CI) |
| interval1 (<130) | 170 | 2.73 (1.81-4.12) | 2.31 (1.46-3.65) | 2.40(1.58-3.65) | 163 | 2.67(1.76-4.07) | 2.23(1.39-3.56) | 2.35(1.53-3.60) |
| interval2 (130-134) | 481 | 1.97(1.45-2.68) | 1.67(1.20-2.34) | 1.80(1.32-2.45) | 454 | 2.00(1.46-2.72) | 1.70(1.21-2.38) | 1.81(1.32-2.48) |
| interval3 (135-140) | 2378 | 1.45(1.17-1.81) | 1.35(1.07-1.72) | 1.40(1.12-1.75) | 2154 | 1.42(1.13-1.79) | 1.32(1.03-1.68) | 1.37(1.09-1.73) |
| interval4(141-145) | 1831 | 1(reference) | 1(reference) | 1(reference) | 1616 | 1(reference) | 1(reference) | 1(reference) |
| interval5(>145)) | 275 | 0.90(0.53-1.52) | 0.82(0.46-1.45) | 0.87(0.51-1.48) | 241 | 0.91(0.53-1.58) | 0.87(0.49-1.55) | 0.89(0.52-1.54) |
| *P* for trend |  | <0.001 | <0.001 | <0.001 |  | <0.001 | <0.001 | <0.001 |
| Serum chloride | n(4966) | Adjusted^a^ OR  (95%CI) | Adjusted^b^ OR  (95%CI) | Adjusted^c^ OR  (95%CI) | n(4479) | Adjusted^a^ OR  (95%CI) | Adjusted^b^ OR  (95%CI) | Adjusted^c^ OR  (95%CI) |
| interval1 (<95) | 381 | 1.65(1.16-2.37) | 1.53(1.03-2.28) | 1.70(1.19-2.44) | 367 | 1.61(1.11-2.32) | 1.51(1.01-2.27) | 1.64(1.14-2.38) |
| interval2 (95-100) | 1297 | 1.19(0.93-1.51) | 1.11(0.85-1.45) | 1.20(0.94-1.53) | 1200 | 1.12(0.88-1.44) | 1.06(0.81-1.39) | 1.14(0.88-1.46) |
| interval3(101-105) | 1964 | 1(reference) | 1(reference) | 1(reference) | 1746 | 1(reference) | 1(reference) | 1(reference) |
| interval4(>105) | 1324 | 1.20(0.93-1.55) | 1.21(0.92-1.60) | 1.18(0.91-1.53) | 1166 | 1.15(0.88-1.49) | 1.15(0.86-1.52) | 1.13(0.86-1.47) |
| *P* for trend |  | 0.125 | 0.354 | 0.068 |  | 0.121 | 0.292 | 0.078 |
| Serum total calcium | n(4143) | Adjusted^a^ OR  (95%CI) | Adjusted^b^ OR  (95%CI) | Adjusted^c^ OR  (95%CI) | n(3740) | Adjusted^a^ OR  (95%CI) | Adjusted^b^ OR  (95%CI) | Adjusted^c^ OR  (95%CI) |
| interval1 (<2.00) | 500 | 0.99(0.69-1.43) | 1.10(0.75-1.62) | 1.01(0.70-1.45) | 453 | 1.02(0.70-1.49) | 1.14(0.77-1.69) | 1.04(0.71-1.51) |
| interval2 (2.00-2.24) | 1872 | 1.14(0.89-1.45) | 1.18(0.91-1.53) | 1.13(0.88-1.43) | 1734 | 1.15(0.89-1.47) | 1.21(0.93-1.58) | 1.14(0.88-1.46) |
| interval3 (2.25-2.58) | 1771 | 1(reference) | 1(reference) | 1(reference) | 1553 | 1(reference) | 1(reference) | 1(reference) |
| *P* for trend |  | 0.742 | 0.862 | 0.831 |  | 0.847 | 0.760 | 0.955 |

OR odds ratio

**Serum potassium:** ^a^Logistic regression model adjusted for age, sex, first admission, smoking, NYHA or Killip functional class, CAD, AMI, hypertension, DM, Stroke/TIA, A fib/A flutter, CKD. heart rate, SBP, DBP, serum sodium, Loop diuretics, ACEI/ARBs, β-blockers, Digitalis, Levosimendan, Nesiritide and Statin.

^b^Logistic regression model further adjusted for not imputed LVEF, eGFR, serum albumin, and log (BNP or NT-pro BNP)

^c^Logistic regression model further adjusted for multiple imputed LVEF, eGFR, serum albumin, and log (BNP or NT-pro BNP)

**Serum sodium:** ^a^Logistic regression model adjusted for age, sex, first admission, smoking, drinking, NYHA or Killip functional class, CAD, AMI, hypertension, DM, Stroke/TIA, A fib/A flutter, CKD, heart rate, SBP, DBP, serum potassium, Loop diuretics, ACEI/ARBs, β-blockers, Digitalis, Levosimendan, Nesiritide, and Statin.

^b^Logistic regression model further adjusted for not imputed LVEF, eGFR, serum albumin, and log (BNP or NT-pro BNP).

^c^Logistic regression model further adjusted for multiple imputed LVEF, eGFR, serum albumin, and log (BNP or NT-pro BNP).

**Serum chloride:** ^a^Logistic regression model adjusted for age, sex, first admission, smoking, drinking, NYHA or Killip functional class, CAD, AMI, hypertension, VHD, DM, Stroke/TIA, A fib/A flutter ,CKD, heart rate, SBP, DBP, serum potassium, serum sodium, Loop diuretics, ACEI/ARBs, β-blockers, MRA, Digitalis, Nitrates, Levosimendan, Nesiritide and Statin.

^b^Logistic regression model further adjusted for not imputed LVEF, eGFR, serum albumin, and log (BNP or NT-pro BNP).

^c^Logistic regression model further adjusted for multiple imputed LVEF, eGFR, serum albumin, and log (BNP or NT-pro BNP).

**Serum total calcium:**  ^a^Logistic regression model adjusted for age, sex, first admission, smoking, NYHA or Killip functional class, CAD, AMI, hypertension, DCM, Stroke/TIA, A fib/A flutter, CKD, heart rate, SBP, DBP, eGFR, serum albumin ,serum potassium, serum sodium, Thiazide diuretics, ACEI/ARBs, β-blockers, MRA, Nitrates, Levosimendan, Nesiritide, and Statin.

^b^Logistic regression model further adjusted for not imputed LVEF and log (BNP or NT-pro BNP).

^c^Logistic regression model further adjusted for multiple imputed LVEF and log (BNP or NT-pro BNP).
